# Supplementary material for: Efficacy, tolerability, and safety of an innovative medical device for improving oral accessibility during oral examination in special-needs patients: A multicentric clinical trial
Source: PLoS One. 2020 Sep 28;15(9):e0239898. doi: 10.1371/journal.pone.0239898 (PMC7521731; doi:10.1371/journal.pone.0239898)
Supplement: S1 Fig — The maximum visibility and probe-ability score for each tooth sector was 2. The maximum Total Oral Accessibility Score was 12. VS, Venham Score. (DOCX) [file pone.0239898.s001.docx]

**S1 Fig Relationship between Venham Score at inclusion and change in Visibility, Probe-ability, and Total Oral Accessibility Scores when the spatula was used.** The maximum Visibility and Probe-ability score for each tooth sector was 2. The maximum Total Oral Accessibility Score was 12. VS, Venham Score.
