# Supplementary material for: Efficacy, tolerability, and safety of an innovative medical device for improving oral accessibility during oral examination in special-needs patients: A multicentric clinical trial
Source: PLoS One. 2020 Sep 28;15(9):e0239898. doi: 10.1371/journal.pone.0239898 (PMC7521731; doi:10.1371/journal.pone.0239898)
Supplement: S1 Table — The conditions underlying these disabilities are indicated below. (DOCX) [file pone.0239898.s002.docx]

**S1 Table The study patients were classified according to whether they had physical, intellectual, and/or behavioral disabilities. The conditions underlying these disabilities are indicated below**

| **Conditions associated with physical disability** | **Conditions associated with intellectual disability** | **Conditions associated with behavioral disability** |
| --- | --- | --- |
| Stroke sequelae | Stroke sequelae | Congenital encephalopathy |
| Drowning sequelae | Alzheimer’s disease | Alzheimer’s disease |
| Cerebral palsy | Cerebral palsy | Cerebral palsy |
| Blindness | Autism spectrum disorder | Autism spectrum disorder |
| Congenital encephalopathy | Congenital encephalopathy | Dravet syndrome |
| Polymalformative syndromes and associated anomalies | Polymalformative syndromes and associated anomalies | Polymalformative syndromes and associated anomalies |
| Richardson’s syndrome | Lewy body dementia | Richardson’s syndrome |
| Huntington's chorea | Microcephaly | Huntington's chorea |
| Monosomy 7 | Monosomy 7 | Monosomy 7 |
| Car accident sequelae | Psychosis | Psychosis |
| Tuberous sclerosis | Joubert syndrome | Tuberous sclerosis |
| Angelman syndrome | Angelman syndrome | Angelman syndrome |
| Goldenhar syndrome | Goldenhar syndrome | Goldenhar syndrome |
| Meningitis sequelae | Kleefstra syndrome | Recklinghausen’s disease |
| Ruptured aneurysm sequelae | Martin-Bell syndrome | Martin-Bell syndrome |
| Rett syndrome | Rett syndrome | CASK-related disorders |
| Trisomy 18,20,22 | Morning glory syndrome | Trisomy 18,20,22 |
| Down’s syndrome | Down’s syndrome | Mowat Wilson syndrome |
| Trisomy 5 | Trisomy 5 | 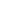 |
