# Supplementary material for: Efficacy, tolerability, and safety of an innovative medical device for improving oral accessibility during oral examination in special-needs patients: A multicentric clinical trial
Source: PLoS One. 2020 Sep 28;15(9):e0239898. doi: 10.1371/journal.pone.0239898 (PMC7521731; doi:10.1371/journal.pone.0239898)
Supplement: S2 File — (DOC) [file pone.0239898.s004.doc]

**Clinical Research Protocol**

Positive opinion from the Ethics Committe of Eastern France III received 07/07/2015

Authorization from the ANSM received 28/11/2014

Version N° 4.0 11/08/2017

Substantially modified compared to version 3.0 07/09/2016

N° IDRCB (*Study Registration Number*): 2012-A01535-38

**EVALUATION OF THE EFFECTIVENESS AND SAFETY OF AN INNOVATIVE DEVICE designed to ENHANCe THE ORAL ACCESSIBILITY OF PEOPLE WITH a handicap who HAVE BEHAVIORAL DISORDERS:**

**THE ORAL ACCESSIBILITY SPATULA (OAS)**

**Sponsor**

CHR of METZ-THIONVILLE

1 allée du château

CS45001

57085 Metz Cedex 03

**Coordinating Investigator**

Dr Daniel ANASTASIO

Department of Odontology

CHR METZ-THIONVILLE – Bel Air Hospital

1-3, rue du Friscaty – BP 60327 – 57 126 THIONVILLE Cedex

Tel: 03 82 55 81 69 / Email: d.anastasio@chr-metz-thionville.fr

**PRINCIPAL CORRESPONDENTS**

**Coordinating Investigator**

Dr Daniel ANASTASIO

Department of Odontology

CHR METZ-THIONVILLE – Bel Air Hospital

1-3, rue du Friscaty – BP 60327 – 57 126 THIONVILLE Cedex

Tel: 03 82 55 81 69 / Email: d.anastasio@chr-metz-thionville.fr

**Associated Investigators**

**Research Methodologist**

Name: Christophe GOETZ

Address: **CHR METZ THIONVILLE**

**Mercy Hospital**

**1 allée du château**

**CS 45001**

**57085 Metz cedex 03**

Tel: 03 87 55 37 46

Email: c.goetz@chr-metz-thionville.fr

**Project Manager**

Name: Nadia OUAMARA

Address: **CHR METZ THIONVILLE**

**Mercy Hospital**

**1 allée du château**

**CS 45001**

**57085 Metz cedex 03**

Tel: 03 87 55 77 52

Email: [n.ouamara@chr-metz-thionville.fr](mailto:n.ouamara@chr-metz-thionville.fr)

**HISTORY OF PROTOCOL UPDATES**

| Version | Date | Reason for update |
| --- | --- | --- |
| V 1.0 | 17/11/2014 | First version |
| V 1.1 | 01/06/2015 | - Change in contacts Sponsor: Project Manager and Clinical Research Associate - Deletion of Annex 10: the budget table - Addition of two additional investigative centers: - CHR Metz Thionville – Mercy Hospital - Centre de Santé Dentaire (*Center for Dental Health*) - Fondation Sonnenhof à Bischwiller (*Sonnenhof Foundation in Bischwiller*) - Update of the provisonal study schedule. |
| V 2.0 | 15/01/2016 | Substantial amendment (SA1): change in the mode of sterilization |
| V 3.0 | 07/09/2016 | Substantial amendment (SA2):   - Substitution of the investigator at the investigative center CHU of Dijon by Dr. Ludwig LOISON-ROBERT   Substantial amendment (SA3):   - Addition of a secondary study objective: operator comfort, which is scored on a numerical scale. |
| V 4.0 | 11/08/2017 | Substantial amendment (SA4):   - Addition of a new investigative center: Centre Hospitalier Emile Durkheim-Epinal (*Emile Durkheim-Epinal Hospital*) and a new principal investigator: Dr. Amélie DALSTEIN - Update in the provisional study schedule: extension of the inclusion period |

**SUMMARY**

**1. PROTOCOL SUMMARY**

**2. sCIENTIFIC Justification AND GENERAL DESCRIPTION OF THE RESEARCH**

**3. RESEARCH OBJECTIVES**

**4. RESEARCH DESIGN**

**5. Sélection AND exclusion OF PEOPLE FROM THE STUDY**

**6. MEDICAL DEVICE**

**7. EVALUATION OF EFFECTIVENESS**

**8. Evaluation OF SAFETY**

**9. StatistiCS**

**10. ACCESS RIGHTs TO DATA AND SOURCE DOCUMENTS**

**11. QUality control and assurance**

**12. ethical and regulatory Considérations**

**13. data processing and retention of documents and data relating to the research**

**14. Financing and insurance**

**15. Rules relating to publication**

**16. List of annexes**

**17. BIBLIOGRAPHIC REFERENCES**

**1.** PROTOCOL SUMMARY

| Title | *OAS: Evaluation of the effectivess and safety of an innovative medical device designed to enhance the oral accessibility of people with a handicap who have behavioral disorders: the Oral Accessibilty Spatula (OAS)* |
| --- | --- |
| Sponsor | *CHR METZ THIONVILLE* |
| Investigator Coordinator | *Dr Daniel ANASTASIO*  *Department of Odontology*  *CHR METZ-THIONVILLE – Bel Air Hospital*  *1-3, rue du Friscaty – BP 60327 – 57 126 THIONVILLE Cedex*  *Tel: 03 82 55 81 69 / Email: d.anastasio@chr-metz-thionville.fr* |
| protocol version | *V4.0 produced 11/08/2017* |
| Justification/context | *It is sometimes difficult, even impossible, to access the oral cavity of people with a behavioral handicap. This is because of their defensive reflexes, where they spontaneously limit the amplitude of the mouth opening or keep their mouth open for very short periods only.*    *The currently available means of keeping the oral cavity open are often unsuitable for these patients and alternative devices have not been described in the literature.*  *This problem makes it difficult to perform preventive oral examinations or provide these patients with oral healthcare.* |
| Main Objective | *Evaluation of oral accessibility during the initial oral examination without and then with the oral accessibility spatula (OAS).* |
| Secondary Objectives | *Evaluation of the safety of the OAS*  *Evaluation of the tolerability of the examination without and with the OAS*  *Evaluation of operator comfort when using the OAS* |
| Primary Endpoint | *Visibility and probe-ability of the three tooth sectors (incisors/canines, premolars, and molars) during the examinations without and with the OAS, as indicated by using an Oral Accessibility Score (this score ranges from 0 = no accessibility to 12 = total accessibility)* |
| Secondary Endpoints | *Identification of adverse events and their frequency.*  *Venham Behavioral Rating Scale*  *Numerical scale measuring operator satisfaction* |
| Méthodology / Study design | *Prospective interventional study, multicenter, open-label*  *Only one visit is planned per patient.* |
| Inclusion Critèria | - *Minor or adult patient with an impairment that is accompanied with behavioral difficulties that are already known or are evinced during the oral examination* - *Venham Scale score of 2 or more when the dentist gradually approaches the oral cavity of the person by first touching their hand, then the lips, then attempting to spread the lips)* - *The holders of parental authority over the minor patients, the legal representatives of the minor or adult patients under guardianship, and the adult patients who are under trusteeship and their trustees are informed about the study and then sign an informed consent form* |
| Exclusion Criteria | - *Pregnant or lactating woman* - *Lack of health insurance* - *Patient is under judicial protection but not yet under guardianship* |
| Procédures | *All patients who present with a behavioral disorder, are being consulted for a preventive dental check or oral care, and who satisfy the eligibility criteria (in particular, they have a Venham Scale score of 2 or more* i.e. *they oppose the dentist’s approach to their oral cavity) are included.*  *The oral accessibility of each eligible patient is then rated by using the oral accessibility score (whic ranges from 0/no accessibility to 12/total accessibility), first without the OAS (*i.e. *the standard examination) and then with the OAS.*  *Adverse events and possible injuries are recorded by completing a chart.*  *The Venham Scale is used again to measure the patient’s behavior during the dental examination without and then with the spatula.*  *At the end of the visit, the operator evaluates his/her satisfaction with the OAS by using a numerical scale (the score ranges from 0: unsatisfactory to 10: very satisfactory).* |
| Number of Patients | *When the OAS is not used, 20% of patients have an oral accessibility score of at least 8.*  *The protocol hypothesizes that the OAS will improve this frequency by 20% (*i.e. *40% of the OAS-treated patients will have an oral accessibility score of at least 8). To validate this hypothesis with an α of 5% and a power of 90%, a total of 140 patients is needed. When potential center-related and investigator-related effects and a study drop out rate of 10% are taken into account, the patient number rises to 200.* |
| Duration of Research | *Only one visit is planned per patient.*  *The total duration of the study therefore corresponds to the recruitment period, which is 33 months.*  *Expected start of the study: March 2016*  *End of recruitment: December 2018*  *End of the study: June 2019* |
| statistical analysis | *The principal analysis will be comparison of the oral accessibility scores during the examination with and without the OAS. Each subject will serve as their own control and the examinations will always be conducted first without the OAS and then with the OAS by the same dental surgeon* |
| Expected results | *The expected result is that the OAS will provide a better quality oral examination without risk to the patient. It is expected that dentists will then be provided with the OAS to improve the therapeutic indications for the use of this tool and the quality of interventions. Thereafter, the OAS will be provided to parents, carers, and nursing staff to facilitate preventive oral healthcare in people with disabilities or dependency.*  ***The expected benefits are therefore as follows:***   - *early detection of oral diseases* - *increased effectiveness of the care provided* - *reduction of the indications for care under general anesthesia* - *facilitating toothbrushing, thereby significantly improving the oral hygiene of these people* - *increasing the prevention of oral diseases by making this spatula available to parents, carers, companions, and healthcare professionals.* |

**List of abbreviations**

| ANSM | Agence Nationale de Sécurité du Médicament et des Produits de Santé (*National Agency for the Safety of Medications and health-related products*) |
| --- | --- |
| CHR | Regional Hospital |
| CHU | University Hospital |
| CNIL | Commission Nationale de l’Informatique et des Libertés (*National Commision for Data Protection and Liberties*) |
| CRA | Clinical Research Associate |
| GCP | Good Clinical Practices |
| ICH | International Conference on Harmonization |
| OAS | Oral Accessibility Spatula |
| SAE | Serious Adverse Event |
| SUSAR | Suspected Unexpected Serious Adverse Reaction |

**2. sCIENTIFIC Justification AND GENERAL DESCRIPTION OF THE RESEARCH**

**2.1. name and description of the experimental medical device**

The present research focuses on an oral accessibility spatula denoted as "OAS".

A device for facilitating oral accessibility can be defined by the following criteria:

- effectively helps to open the mouth
- easy to use
- helps the healthcare team to maintain distance from the mouth opening
- aids oral examinations, and possibly toothbrushing and dental care.

The OAS is an **innovative** medical device that aids access to the oral cavity.

The overall objective is the creation of a tool that facilitates oral accessibility in people with a handicap or dependency whose self-protective reactions make it difficult for the dentist to access the mouth during oral examination. This tool is specifically designed for people with behavioral difficulties due to their impairment.

The OAS meets the following criteria:

- shape adapted for the oral cavity of both adults and children
- non-traumatic for the dental structures
- non-traumatic for the mucosal tissues
- easy to use
- good grip
- hygienic, can be cleaned, can be sterilized according to hospital standards (one single sterilization before use, the spatula is intended for single use only)

It is a device that provides technical aid during preventive or curative management. It is not a therapeutic device.

Moreover, this device is not suitable for people who present with a pathological limitation in mouth opening.

**2.2. Summary of the results of available non-clinical and clinical trials that relate to the present research**

There is currently no medical device of this type.

This spatula is novel in that it allows access to the oral cavity and then holds the mouth open during the examination.

This device is more suitable than the classical intraoral mouth gags, which are more difficult to insert into the mouth and keep between the dental arches during the examination.

The current devices that are used to aid oral accessibility are:

- metal mouth gags
- Intraoral rubber mouth props
- spinning top-shaped devices

None of these devices satisfactorily meet the criteria for oral accessibility.

Because of its innovative nature, the OAS has been the subject of a patent application file dated 29/03/2010. The patent was issued on 08/11/2013.

**2.3. Summary of the anticipated and known benefits for and risks to the people participating in the study**

**Benefits for the patient:**

- better accessibility to the oral cavity
- patient can be screened for carious and peridontal lesions
- possible pain from proven lesions can be managed
- the patient’s level of oral hygiene can be assessed
- diagnosis of oral diseases
- a treatment plan that is adapted to the nature of the impairment and the degree of cooperation can be established

**Adverse effects:**

- possible oral lesions (see section 4.4.2).
- inhalation or ingestion of a tooth or tooth fragment
- there are no other study-related risks for people included in the protocol.

The safety of the device will be evaluated during the study

**2.4. Description and justification of the terms of use and the duration of treatment**

The oral management of people with a behavioral impairment is complicated by their instinctive self-protective reactions.

The oral cavity is a unique area in that it is both the aerodigestive junction (where both alimentation and respiration start) and the seat of oral expression.

The self-protective behavior involves withdrawal with forward bending of the head and closure of the mouth.

The advantage of the OAS is that it overcomes the self-protective mouth closure since it can be efficiently inserted into the mouth, thereby facilitating the opening of the mouth.

The duration of treatment is not addressed because the effectiveness of the device will be evaluated during a simple oral examination.

**2.5. Statement that the research will be conducted in accordance with the protocol, GCP, and current regulations and legislation**

The research will be conducted according to the protocol, Good Clinical Practice, and the current regulations.

**2.6. Description of the study population**

The study population consists of adult and/or minor patients who present with a behavioral disorder and who are:

- referred to the Department of Odontology for a preventive assessment or oral healthcare (whatever it may be)
- are cared for as part of an annual oral screening visit within the specialized institution on which the patient depends

The study population therefore consists of people who have a particularly protected status in terms of biomedical research (Articles L1121-8 and L1121-7 of the Code de la Santé Publique [*French Public Health Code*]). In other words, the adult patients are subject to a legal protection order as well as the minor patients.

**3. research Objectives**

**3.1. Primary Objective**

The primary objective of our study is the evaluation of oral accessbility during an initial oral examination, without and then with the OAS.

**3.2. Secondary objectives**

The secondary objectives are:

- Assessment of the safety of the spatula
- Assessment of the tolerability of the spatula, without and then with the spatula.
- Assessment of the comfort of the operator

**3.3. Objectives of any possible ancillary study**

**Not applicable**

**4. Research design**

**4.1. assessment methods**

- *Primary assessment method*

The primary assessment is the visibility and probe-ability of the incisor/canine, premolar, and molar tooth sectors. This assessment is made during a vestibular and palatal/lingua approach.

The visibility and probe-ability are scored so that an "oral accessibility score" that ranges between 0 (no accessibility) and 12 (total accessibility) can be calculated. How this score is calculated is described in section 4.4.1.

- *Secondary assessment methods*

The safety of the spatula will be evaluated by recording the adverse events and determining their frequencies.

The tolerability of the examination, without and then with the OAS, will be evaluated by using the validated Venham Behavioral Rating Scale.

The comfort of the operator during the use of the OAS spatula will be evaluated at the end of each visit by the operator using the OAS, by applying a numerical satisfaction scale whose scores range from 0 (unsatisfactory) to 10 (very satisfactory).

## 4.2. research méthodology

The patients will be recuited:

- either in the Department of Odontology, as part of a preventive assessment or for oral healthcare (whatever it may be)
- or in the specialized institution on which the patient depends, as part of annual oral screening.

For all patients, the assessment will be conducted in one visit.

The study population consists of people who have a particularly protected status in terms of biomedical research. In other words, the adult patients are subject to a legal protection order as well as the minor patients.

The patients will therefore receive information from the investigator that is tailored to their ability to understand.

The information will also be given:

- to the holders of parental authority over the minors
- the legal representatives of minors and adults under guardianship
- the trustees for adults under trusteeship

The informed consent form will be signed:

- by the holders of parental authority over the minors
- by the legal representatives of the minors and adults under guardianship
- by the trustees of the adults under trusteeship

The personal commitment of the patients themselves will always be sought, **as much as possible**, and their refusal cannot be overruled.

Once the consent form has been signed, the investigator will evaluate whether the patient meets the inclusion criteria: the Venhman Scale score will be calculated during a gradual approach to the oral cavity of the person (first touching the hand, then the lips, then attempting to spread the lips). The patient is included if the Venham score is 2 or above, meaning there is opposition to the examination.

If the patient meets all the inclusion criteria and does not meet any of the exclusion criteria, the investigator will proceed to the first examination of the oral cavity **without** the OAS.

He/she will measure the oral accessibility scores and any problems (see section 4.4)

He/she will also calculate the Venham Scale score during the examination without the OAS (evaluation of patient tolerance).

In a second step, the investigator will examine the oral cavity **with** the OAS.

He/she will measure the oral accessibility scores and any problems (see section 4.4)

He/she will also calculate the Venham Scale score during the examination with the OAS (evaluation of patient tolerance).

Finally, after performing both examinations (without and then with the OAS), the operator will evaluate his/her level of comfort when using the OAS spatula compared to the usual examination without the OAS.

## 4.3. Description of the measures taken to reduce and avoid bias

Each patient will their own control.

The examinations will always be done first with the OAS and then with the OAS because it is not desirable to perform an examination that starts with the use of a constraining device. The examination without and then with the OAS will be performed by the same dental surgeon to maximize patient comfort (because inflicting two different operators on the patient could be disturbing and changing operators could prolong the examination).

This design makes it possible to neutralize the operator effect, but it can introduce possible evaluation bias because the same dental surgeon makes the two 2 assessments. However, the only design that would address this bias would be a parallel-arm trial where one arm is examined with the OAS only (*i.e.* there is no initial approach without the OAS). This is undesirable for the patient. To minimize this bias as much as possible, oral accessibility will be assessed with an objective score that involves counting the visible areas of the oral cavity (see 4.4.1). In addition, all investigators will be made aware of the importance of strictly adhering to this score to avoid evaluation bias.

The examination with the OAS will always follow an examination without the OAS, which runs the risk of habituation bias. However, this bias cannot be limited because an examination of the oral cavity must never start with the use of the OAS

To avoid any conflict of interest, we specify that the coordinating investigator of this study, who is also the inventor of the OAS, can be an inclusion operator but cannot perform examinations of the oral cavity to measure oral accessibility.

This study will be conducted as an open-label study because the nature of the study device means that neither the patient nor the investigator can be blinded.

## 4.4. the medical device being studied

## 4.4.1 oral accessibility score variables

The following variables will be evaluated without and then with the OAS (note that a "tooth sector" refers to the indicated teeth on both the maxilla and the mandible):

- intraoral visibility of the left and right incisor/canine tooth sectors
- intraoral visibility of the left and right premolar tooth sectors
- intraoral visibility of the left and right molar tooth sectors
- probe-ability of the left and right incisor/canine tooth sectors
- probe-ability of the left and right premolar tooth sectors
- probe-ability of the left and right molar tooth sectors

Each item is scored from 0 to 2, where:

- 0 = negative result for both the left and right sides of a specific tooth sector
- 1 = positive result for either the left or right side (but not both) of a specific tooth sector
- 2 = positive result for both the left and right sides of a specific tooth sector

The oral accessibility score during the examination without the OAS will be scored from 0 to 12.

The oral accessibility score during the examination with the OAS will be scored from 0 to 12.

## variables for assessing OAS-related adverse events

11 items are used to evaluate the safety of the OAS. These adverse events are described as often occurring when intraoral mouth props, metal mouth gags, and spinning top-shaped devices are used:

- labial injuries
- gingival injuries
- cheek injuries
- lingual injuries
- dental fracture
- dental luxation
- dental expulsion
- nausea/vomiting
- pain
- joint complications
- others

Each item with be scored 1 (yes) or 0 (no).

## 4.5. Expected duration of study subject participation and description of the chronology and duration of all study periods

Duration which which each patient will participate: 1 visit (approximately 30 minutes)

Duration of patient recruitment: 33 months

Total duration of the research: 39 months

Total forecast duration of the research (duration of both inclusion and participation): 39 months

The patient only participates in one visit:

- signing of consent by the patients’ representative if they are a minor or adult under guardianship, by the patients themselves if they are adults under trusteeship

- verification of the eligibility criteria

Examination of the oral cavity **without** the OAS:

- evaluation of accessibility and possible adverse events (see section 4.4)

- score obtained with the Venham Scale during the examination without the OAS.

Examination of the oral cavity **with** the OAS:

- evaluation of accessibility and possible adverse events (see section 4.4)

- score obtained with the Venham Scale during the examination with the OAS.

The operator finishes each visit by using a numerical scale to evaluate how comfortable the OAS spatula was to use.

There is no study-specific follow-up at the end of this visit.

**4.6. Measures to maintain or lift blinding, if appropriate.**

Not applicable, the study is an open-blind study

**4.7. Identification of all data that will be collected directly in case report forms, which will be considered to be source data.**

Not applicable

**4.8. biological sample collection**

Not applicable

**5. Sélection and exclusion of study subjects**

The study population consists of people who have a particularly protected status in terms of biomedical research. In other words, the adult patients are subject to a legal protection order as well as the minor patients.

Research on these populations is justified because:

- it responds to the health needs and priorities of these populations

- the results of the research will be beneficial for these populations

- the study cannot be conducted on another population

- the risks and disadvantages are minor in terms of the expected benefits.

In addition, it is necessary to test the effectiveness and safety of the spatula on minors to confirm that the OAS is suitable for small oral cavities.

**5.1. Inclusion criteria**

- Minor or adult patient with am impairment accompanied with behavioral difficulties (known beforehand or discovered during oral examination)
- Score of 2 or more on the Venham Scale on a gradual approach to the oral cavity of the person (defined as touching the hand, then the lips, then attempting to spread the lips)
- The study-specific informed consent form is signed after the holders of parental authority over minor patients, the legal representatives of the minors and adults under guardianship, and the adult patients under trusteeship and their trustees are given information specific to the study

**5.2. exCluSion criteria**

- Pregnant or lactating woman
- Lack of health insurance
- Patient is under judicial protection but not yet under guardianship

**5.3. Procédure for premature STopping of the study or exclusion (stopping treatment + stopping follow-up)**

**5.3.1. Criteria and procedures for stopping treatment/medical device use and exclusion**

- *Premature stop criteria*

It is impossible to perform the evaluations, without and then with the spatula, due to patient behavior that involves their physical integrity (score of 5 on the Venham Scale).

- *Follow-up Methods for persons excluded from the study*

Patients with a score of 5 on the Venham Scale will be examined outside the study by using the following medical techniques:

- sedated by inhalation of an equimolar mixture of oxygen and nitrous oxide
- under sedation using Midazolam (intra-rectal or intravenous)
- Under general anesthesia if necessary.

**5.3.2. data collection and follow-up procedures**

The data will be collected in paper case report forms, from the source file of the

patient.

Patients only participate in one visit as part of the study. There is no follow-up planned.

**5.3.3. Procedure for of replacing people**

The number of subjects required is based on a 10% examination drop-out rate. Patients who have dropped out of the study will not be replaced.

.

**5.3.4 Follow-up procedure**

Not applicable.

**5.4. recruitment procedure**

The patients will be recruited:

- either in the Department of Odontology, as part of a preventive assessment or for oral healthcare (whatever it may be)
- or in the specialized institution on which the patient depends, as part of annual oral screening.

The inclusion period will be 33 months.

**6. medical device**

**6.1. medical device used**

*Figure 1: photo of the Oral Accessibility Spatula OAS*


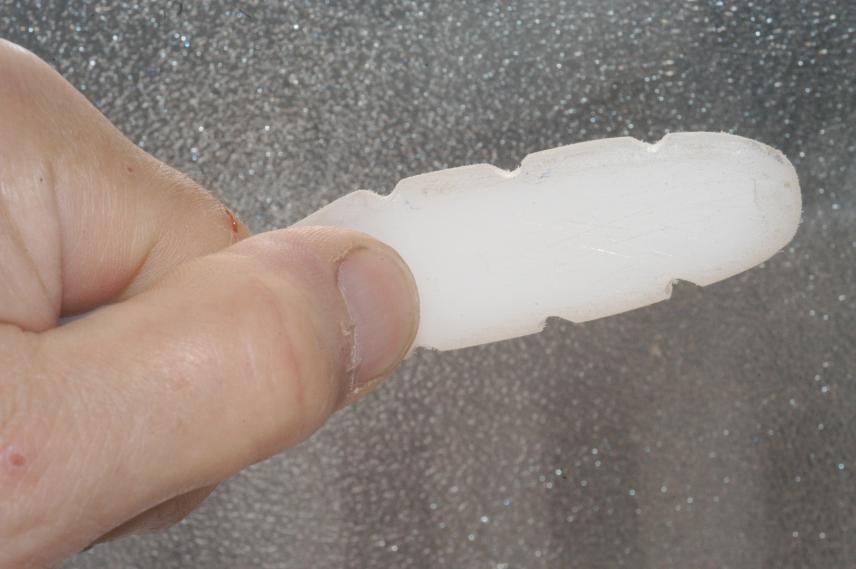


## *Procedures employed when using the OAS.*

The OAS consists of a blade and a handle (see Appendices 1, 2 and 3). The shape of the blade facilitates its insertion into the oral cavity between the dental arches.

Rotating the handle makes it possible to exert sufficient force to cause the mouth to open.

The blade bears notches in which the maxillary and mandibular teeth can be wedged, thus wedging open the arches during the oral examination.

The spatula is made entirely of transparent polypropylene Bormed ™ RF830MO. This prevents trauma to the dental elements in case the patient bites down on the spatula.

The shape of the blade was carefully designed to prevent damage to the various tissues in the oral cavity.

The material is compatible with one sterilization according to current hospital standards: one sterilization with ionizing radiation from gamma rays (25 kGy).

The polypropylene Bormed ™ RF830MO can be sterilized by gamma radiation with one 25kGy dose. This mode of sterilization means the material will be sterile for 5 years if it is stored at ambient temperature. (See data sheet in Annex 5)

The users of this spatula during the clinical trial are dental surgeons who are accustomed to accessing the oral cavity.

The use of the OAS does not require, *a priori*, training of the investigators. However, instructions for its use have been produced (Annex 4) so that the technique for using the medical device is standardized. The users will be asked to carefully respect the the physical integrity of the person when using the spatula to access their oral cavity.

During the clinical trial, the OAS will be sterilized according to current hospital standards and packaged in individual sterile double packaging. They all come from the same manufacturing batch to ensure that they are completely identical. Each spatula will be disposed of after use and destroyed like any medical waste.

**6.2. Description of any medication, medical device (other than the experimental medication/medical device) that will be used for the research needs**

Not applicable

**6.3 Médications and treatments that are authorized and prohibited during the study**

- No medical device (other than the OAS) will be used to help open the oral cavity during the study.
- No sedative medication will be used outside the current treatments.

**6.4. Méthod for monitoring compliance**

The OAS is not a therapeutic device, rather, it is a device that provides technical assistance during preventive or curative management. Each patient only participates in one visit. Therefore, there will be no monitoring of patient compliance.

**7. EVALUATION OF EFFICACY**

## 7.1. Description of the efficacy variables

The data that will be collected to evaluate the effectiveness of the OAS are described in section 4.4.1

## 7.2. Planned methods and schedule for measuring, collecting and analyzing the efficacy variables.

The methods and schedule for measuring, collecting and analyzing these variables are described in section 4.5.

For all patients, effectiveness will be measured at the single study visit.

**8. Evaluation OF SAFETY:**

**8.1. Description of the safety variables**

The data collected as part of patient monitoring are described in section 4.4.2.

**8.2. Planned methods and schedule for measuring, collecting and analyzing the safety variables**

Each patient is evaluated once in this study. The safety variables will be identified during this visit.

**8.3 Procédures for the registration and notification of adverse events**

**8.3.1 Definitions**

An **adverse event** is any untoward medical occurrence in a person who participates in biomedical research, regardless of whether this event is causally linked to the research or the study device.

An **adverse reaction** to a medical device is any adverse and unwanted reaction to the medical device or any incident that could have caused this reaction if an action had not been carried out, in a person who participates in research or in the user of the medical device; or any reaction that relates to failure of the medical device that is harmful to the health of the study subject.

A **serious adverse event (SAE) or serious adverse reaction** is an event or a reaction that is life-threatening; may have contributed to the death, hospitalization, or prolongation of hospitalization of the patient; contributed to incapacitation or a significant or long-lasting disability; resulted in a birth defect or congenital malformation; or any event deemed significant by the investigator.

**The list of expected adverse events** in the study is based on the reference document.

An SAE is considered to be a suspected unexpected serious adverse reaction (SUSAR) when it is not on this list.

**A new fact** can be: unexpected frequency of an expected SAE, an SAE that relates to the test procedure, insufficient efficacy in life-threatening diseases, non-clinical data.

**8.3.2 Reference document for defining SAEs**

There is no reference document for the experimental medical device under study, since it is a new medical device.

An investigator brochure has been produced by Dr. Anastasio, the inventor of the spatula.

The expected events are those that are frequently encountered when using intraoral rubber mouth props, metal gags, or spinning top-shaped devices.

**8.3.3 List of expected adverse reactions**

Adverse reactions that relate to the use of the experimental medical device are currently not known because it is a new medical device.

The expected adverse events in the list for evaluating spatula-related incidents are:

- labial injuries
- gingival injuries
- cheek injuries
- lingual injuries
- dental fracture
- dental luxation
- dental expulsion
- nausea/vomiting
- pain
- joint complications
- others

Expected SAEs are inhalation or ingestion of a tooth or a tooth fragment that requires hospitalization.

Inhalation or ingestion of a tooth or tooth fragment may occur as a result of dental dislocation or fracture, events occurring on a tooth that moved before the examination was performed.

A maximum of 1 to 2 OAS-related SAEs is expected for all patients included in the study.

**8.3.4 Notification of SAEs or new facts**

As soon as an investigator becomes aware of an SAE or a new fact, he/she will notify the sponsor without delay by faxing the SAE declaration form (Annex 7).

- If it is an suspected unexpected serious adverse reaction (SUSAR), the sponsor will contact the investigator to write an initial report that will be sent to ANSM, the ethics committee, and the coordinating investigator within 7 days (if the SUSAR is life-threatening or the patient has died) or 15 days (in other cases).

If the event is not resolved by the date the fax is sent, the investigator is required to send an supplementary report (within 8 days in the event of death or a life-threatening SUSAR; within 15 days in other cases) to document progress in the case or to update missing data.

- If it is an SAE that is linked to the use of the experimental medical device, the declaration will be sent to the ANSM as described above and to the ethics committee every quarter.
- If it is a new fact, the sponsor will declare it to the ANSM and the ethics committee within a maximum of 15 days; additional information can be provided within an additional 15 days.
- If it is an expected SAE, it will be recorded by the sponsor and reported in the annual safety reports.

**8.3.5 Notification of non-serious adverse reactions**

They will be described briefly by the investigator in the case report form in the section dedicated to adverse events.

**8.4. Procedures and duration of follow-up of persons with adverse events**

When a serious adverse event persists, even after the end of the study, the investigator will follow the patient until the event is considered resolved. The follow-up data will be sent to the sponsor.

**8.5. Committees specific to the research (varies depending on the protocol)**

**8.5.1 Steering committee**

It will consist of the initiating clinician of the project, the biostatistician in charge of the project and the representatives of the sponsor.

It will define the general organization and conduct of the research and will coordinate the information.

It will initially determine the methodology; will decide what do do in unforseen cases during the course of the research; and will monitor the progress of the research, particularly regarding patient tolerance and adverse events.

### 8.5.2 Independent monitoring committee

The nature of this study does not justify the establishment of an independent monitoring committee.

### 8.5.3 Independent committee for evaluating critical events

The nature of this study does not justify the establishment of an independent committee that evaluates critical events.

**8.6 Safety reports**

- Annual safety reports: the sponsor drafts the annual safety reports and sends them to the ANSM, the ethics committee, and the coordinating investigator. The coordinating investigator will send the sponsor all of the data needed to draft these reports.
- Final report: it is written by the sponsor and the coordinating investigator within a one year of the end of the study. All investigators will be informed of the results of the study.

A summary is sent to the ANSM by the sponsor.

**9. StatistiCS**

**9.1. description of the planned statistical méthods, including the schedule for planned intermediate analyses**

The main analysis will consist of

1. Describing the sample to assess the necessity and feasibility of using the OAS,

2. Checking the inclusion criteria and the representativeness of the final sample,

3. Comparing the examinations with and without the OAS in terms of the frequency of patients whose oral accessibility score was at least 8 by using a McNemar test, and the average oral accessibility scores by using Student's Paired *t-*test. The data will also be examined for possible center and investigator effects.

4. Evaluate the safety of the OAS by quantifying and describing the observed adverse events.

**9.2. Number of people to include in the research and the statistical justification**

Without the OAS, 20% of the patients will have an oral accessibility score of at least 8.

The protocol hypothesizes that the use of the OAS will improves this frequency by 20% (*i.e*. 40% of patients will have a score of at least 8). To validate this hypothesis with an α of 5% and a power of 90%, a total of 140 patients is needed. When potential center-related and investigator-related effects and a study drop out rate of 10% are taken into account, the patient number rises to 200.

**A feasibility study** was conducted in the Department of Odontology of the CHR METZ-THIONVILLE over a period of 12 months (January to December 2009). During this period, 104 patients meeting the inclusion criteria of our study were aided by the Department of Odontology, which confirms the feasibility of the recruitment target that was set.

**9.3. degrée of planned statistical significance**

The α is fixed at 5%

**9.4. Statistical criteria for stopping the research**

None.

**9.5. méthod for taking into account missing, discarded, or invalid data**

Subjects without complete data will not be included in the comparative analysis of the oral accessibility scores. They will be described and compared to the subjects with complete data.

**9.6. management of amendments to the original analysis plan**

No special management is foreseen.

Any amendments must be the subject of an explicit discussion between the investigator and the methodologist.

**9.7. choice of people to include in the analyses.**

Subjects without complete data will not be included in the comparative analysis of the oral accessibility scores. They will be described and compared to subjects with complete data.

**10. ACCESS rights TO DATA AND SOURCE DOCUMENTS**

All data and information concerning the patient will remain strictly confidential. The persons with direct access in accordance with the current laws and regulations, in particular Articles L.1121-3 and R.5121-13 of the Public Health Code (for example, investigators, those in charge of quality control, the monitors, the clinical research assistants, the auditors, and all people who collaborate in the trials), will take all necessary precautions to ensure the confidentiality of information relating to the investigational drugs, the trials, and the persons who participate (particularly with regard to their identity and the results that were obtained). The data collected by these people during quality controls or audits are then made anonymous.

**11. QUALITY Contrôl and assurance**

**11.1 Monitoring**

The risk level assigned to the study, which is defined on the basis of the OPTIMON table (Appendix 8), is B (Class I device not marked with CE). According to the Logistics-Impact-Resources score of the DRCI's "Quality of Promotion" working group (Annex 9), a MINIMAL level of monitoring will be necessary.

The Clinical Research Assistant who represents the sponsor will visit the investigative center at the pace of MINIMAL level monitoring:

- Site-opening visit: this will occur before the 1st inclusion to set up the protocol and become familiar with the various stakeholders participating in the biomedical research.

- During subsequent visits, only consent will be monitored. If consent is non-compliant, the patient records will be monitored randomly at the site.

The sheets of the case report forms will be separated and retrieved by the Clinical Research Associate.

The principal investigator as well as other investigators who include or monitor the study participants commit to meeting the Clinical Research Associate at regular intervals.

During these site visits, and in accordance with Good Clinical Practices, the following elements will be reviewed:

- Compliance with the protocol and the procedures in the research,
- Examination of the source documents: notification that the patient file contains a signature indicating the patient's consent or the consent of the holders of parental authority or the patient’s guardian.

- Closing visit: retrieval of the last sheets from the case report forms and the inventory records of the pharmacist and archiving of the biomedical research documents.

As such, the investigator agrees to make the following available to the Clinical Research Associate during his/her monitoring visits:

⮚ The medical records of the patients

⮚ The case report forms

⮚ The forms that include the consent of the patients.

### 11.2 Completion of data in the case report form

All information required by the protocol must be provided in the case report form and the investigator must provide an explanation for each missing data point.

The data will have to be transferred to the case report forms as they are obtained, regardless of whether they are clinical or paraclinical data. The data will have to be copied into the case report forms with clear and legible writing in black ink (to facilitate duplication and computerization).

The erroneous data found in the case report forms will be crossed out clearly and the new data will be copied into the case report form along with the date and initials of the investigative team member who made the correction.

The anonymity of the subjects will be ensured by a code number and the research participant’s initials on all research documents, or by redacting by appropriate means the personal data of the patients on the copies of the source documents that will be used for research documentation.

The computerized data will be placed in a file and declared to the CNIL according to the procedure adapted to each case.

**12. ETHICAL AND REGULATORY Considérations**

The sponsor and the investigators agree to ensure that this research is conducted in compliance with Law 2004-806 of 9 August 2004 and in agreement with Good Clinical Practices (ICH version 4 produced on 1 May 1996 and settled on 24 November 2006) and the Declaration of Helsinki (Ethical Principles for Medical Research on Human Subjects, Seoul 2008).

The research is conducted in accordance with the present protocol. The investigators commit to adhering to the protocol in all respects, especially with regard to collecting consent and notifying the authorities and following up serious adverse events.

Each investigator will agree to respect the obligations of the law and to conduct the research according to Good Clinical Practices amd the terms of the current Declaration of Helsinki.

12.1 Request for authorization from the ANSM

To be able to start the research, the sponsor must submit an application file for authorization by the competent authority, namely, the ANSM. The competent authority defined in Article L.1123-12 makes decisions regarding the safety of persons who participate in biomedical  research. In particular, they assess the quality and safety of the products that are used during the research and ensure that, where applicable, they are used according to current standards and their terms of use. The competent authority also assesses the safety of persons with regard to the acts performed, the methods used, and the planned monitoring procedures in the study.

12.2 Request for advice from the Ethics Committee

In accordance with Article L.1123-6 of the Public Health Code, the research protocol must be submitted by the sponsor to an Ethics Committee. The sponsor will inform the competent authority about the opinion of the Ethics Committee before the start of the research.

12.3 Amendments

The amendments must be categorized as substantial or not substantial.

A substantial amendment is a change that could, in one way or another, modify the guarantees to the persons who participate in the biomedical research (modification of an inclusion criterion, extension of the inclusion period, participation of new centers, ...).

After beginning the research, any substantial amendment by the sponsor must obtain, before its implementation, a favorable opinion and authorization from the competent authority. In this case, and if it is deemed necessary, the committee will ensure that the people involved in the research provide new consent.

In all cases of substantial amendment, the sponsor must request authorization from the ANSM and/or request an opinion from the Ethics Committee.

12.4 CNIL déclaration

This research falls within the framework of "Reference Methodology" (MR-001) in accordance with the provisions of Article 54 (paragraph 5) of the modified Law of 6 January 1978 that relates to data processing and liberties. This modification was approved by the decision of 5 January 2006. The CHR of Metz-Thionville signed a commitment to comply with "Reference Methodology".

Only the data needed for the research will be collected. The patient nevertheless has the right to oppose the automated processing of the data concerning him/her. The patient will have the right to access the data concerning him/her at any time. He/she will also have the right to request the rectification of inaccurate data or data that has become inaccurate. He/she will be able to exercise their rights with Dr ANASTASIO, the coordinating investigator.

For all information of a medical nature, the rights of the patients may be exercised directly or *via* the doctor of their choice.

In the case where the consent was given by the family (or authorized person), only the patient is able to access the data concerning him/ her as described above.

**12.5 Participant information sheet and Informed consent**

The study population consists of people who have a particularly protected status in terms of biomedical research. In other words, the adult patients are subject to a legal protection order as well as the minor patients.

- **For adult patients under guardianship and minor patients:**

Before obtaining the consent of the legal representative or the holders of parental authority over the patient, the investigator agrees to provide the patient with information on the proposed study that is as clear, honest, appropriate, and complete as possible; the investigator also gives the patient a participant information sheet that is adapted to their ability to understand.

The information is also delivered:

- To the holders of parental authority over minors
- the legal representatives of minors and adults who are under guardianship

The information relates to:

- the objectives and limitations of the study for the patient
- the right of the legal representative to refuse to have the patient participate in the study
- the right of the legal representative to have the patient leave the study at any time

When the substance of the information has been given to the patient and the legal representative of the patient and when the investigator has ensured that the latter understands the implications of the participation of the patient in the trial, the written consent of the legal representative will be collected by the investigator. The patient's personal commitment will always be sought.

Two original copies of the consent form will be signed by the holders of parental authority or the guardian and the investigating doctor:

⮚ A copy will be given to the holders of parental authority or the guardian, as the case may be

⮚ One copy will be kept and archived by the investigator.

- **For adult patients under trusteeship**:

The patient, assisted by his/her trustee, will receive oral and written information that is clear, honest, complete and adapted to his/her ability to understand.

If the patient agrees to participate in the study after having had time to reflect and having had the opportunity to ask all the questions he/she wishes, the patient, with the assistance of his/her trustee, may sign his/her own consent form in two original copies. A copy will be given to the patient and the 2^nd^ will be kept and archived by the investigator.

### 12.6 Final study report

The final report of the research will be written by the coordinator in collaboration with the biostatistician for this research. This report will be submitted to each investigator to solicit their opinion. Once a consensus has been reached, the final version must be endorsed by the signature of each of the investigators and sent to the sponsor as soon as possible after the effective end of the research. A report drawn up in accordance with the reference plan of the competent authority must be sent to the competent authority and the Ethics Committee within one year of the end of the research, namely, the last follow-up visit of the last included study participant. In the case of premature termination of the research, the report should be sent in 90 days.

13. DATA PROCESSING AND RETENTION OF DOCUMENTS AND DATA RELATING TO THE RESEARCH

The documents from research that falls within the scope of the law on biomedical research must be archived by all parties for a period of 15 years after the end of the research*.*

This indexed archive contains:

- The copies of the authorization letters from the ANSM and the mandatory notification of the Ethics Committee
- The successive versions of the protocol (identified by the version number and the date of the version),
- The letters of correspondence with the sponsor,
- The signed consent forms, in sealed envelopes, from the subjects (signed by the holders of parental authority in the case of minor subjects, signed by the guardian in the case of patients under guardianship) with the matching inclusion list or register,
- The completed and validated case report form of each included subject,
- All study-specific appendices,
- The final study report arising from the statistical analysis and quality control of the study (sent twice to the sponsor).
- The certificates of any audits that were performed during the research

The database that gave rise to the statistical analysis must also be archived by the person in charge of the analysis (paper or computer).

**14. FinancING AND INSURance**

**14.1 Insurance**

The sponsor has taken out an insurance policy with SHAM that runs for the duration of the study and covers its own liability and that of any stakeholder involved in the study regardless of the nature of the relationship between the stakeholders and the sponsor.

### financing

The first funding was obtained in 2008: this was a sum of € 3,000 that was paid by la Mutuelle Nationale des Hospitaliers (a national supplementary mutual health insurance for hospitals) after the 11^th^ award for "Innovations in Handicap" was won by the Department of Odontology of the Bel-Air Hospital.

Additional financing for the realization of the study was obtained by the CHR Metz Thionville under the framework of the PHRC-I 2011 (the 2011 interregional clinical research program for hospitals).

OAS spatulas will be manufactured by a company called PrecisLux.

500 spatulas will be provided, all from the same lot.

**15. RULES PERTAINING TO publication**

The CHR of Metz-Thionville is the owner of the data and it cannot be used or transmitted to a third party without its prior consent.

Where appropriate, the CHR of Metz-Thionville must be mentioned as the sponsor and the financial supporter of the biomedical research.

**16. List og the annexs**

Annex 1: The Oral Accessibility Spatula: illustration

Annex 2: Patent drawing of the Oral Accessibility Spatula

Annex 3: The Oral Accessibility Spatula: Illustration showing how to use it

Annex 4: Operating instructions for the Oral Accessibility Spatula

Annex 5: Data sheet for the polypropylene Bormed™ RF830MO

Annex 6: Venham Scale

Annex 7: SAE declaration form

Annex 8: OPTIMON table

Annex 9: LIR Score.

##

**17. bibliographic REFERENCES**

**Collado V, Faulks D, Hennequin M**. A survey of the difficulties encountered during routine hygiene and health care by persons with special needs. *Disabil Rehabil. 2008; 30(14):1047-54*.

**Davis MJ**. Issues in access to oral heath care for special care patients. *Dent Clin North Am. 2009; 53(2):169-81.*

**Glassman P**. A review of guidelines for sedation, anaesthesia, and alternative interventions for people with special needs. *Spec Care Dentist. 2009; 29(1):9-16*.

**Christensen GJ**. Special oral hygiene and preventive care for special needs. *J Am Assoc. 136;8:1141-1143.*

**Hennequin M, Moysan V, Jourdan D, Dorin M, Nicolas E**. Inequalities in oral heath for health for children with disabilities: a french national survey in special schools. *PLoS One. 2008;3(6):e2564.*

**De Jongh A, Van Houtem C, Van der Schoof M, Resida G, Broers D**. Oral health status, treatment needs, and obstacles to dental care among noninstitutionalized children with severe mental disabilities in the Netherlands. *Special Care Dentist. 2008;28(3):111-5*.

**Marchall J, Sheller B, William BJ, Mancl L, Cowan C**. Cooperation predictors for dental patients with autism. *Pediatr Dent. 2007;29(5):369-376*.

**Nussbaum BL**. Dental care for patients who are unable to open their mouths. *Dent Clin North Am. 2009;53(2):323-8.*

**Romer M**. Consent, restraint, and people with special needs: a review. *Spec Care Dentist. 2009 ;29(1):58-66.*

**Annex 1: ORAL ACCESSIBILIty SpatulA (OAS)**

**illustration**


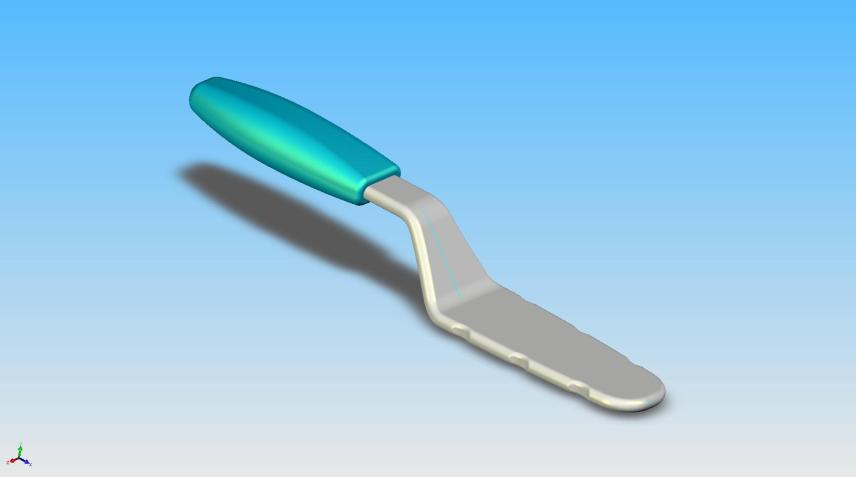


**Annex 2: PATENT DRAWING OF THE ORAL ACCESSIBILITY spatula**
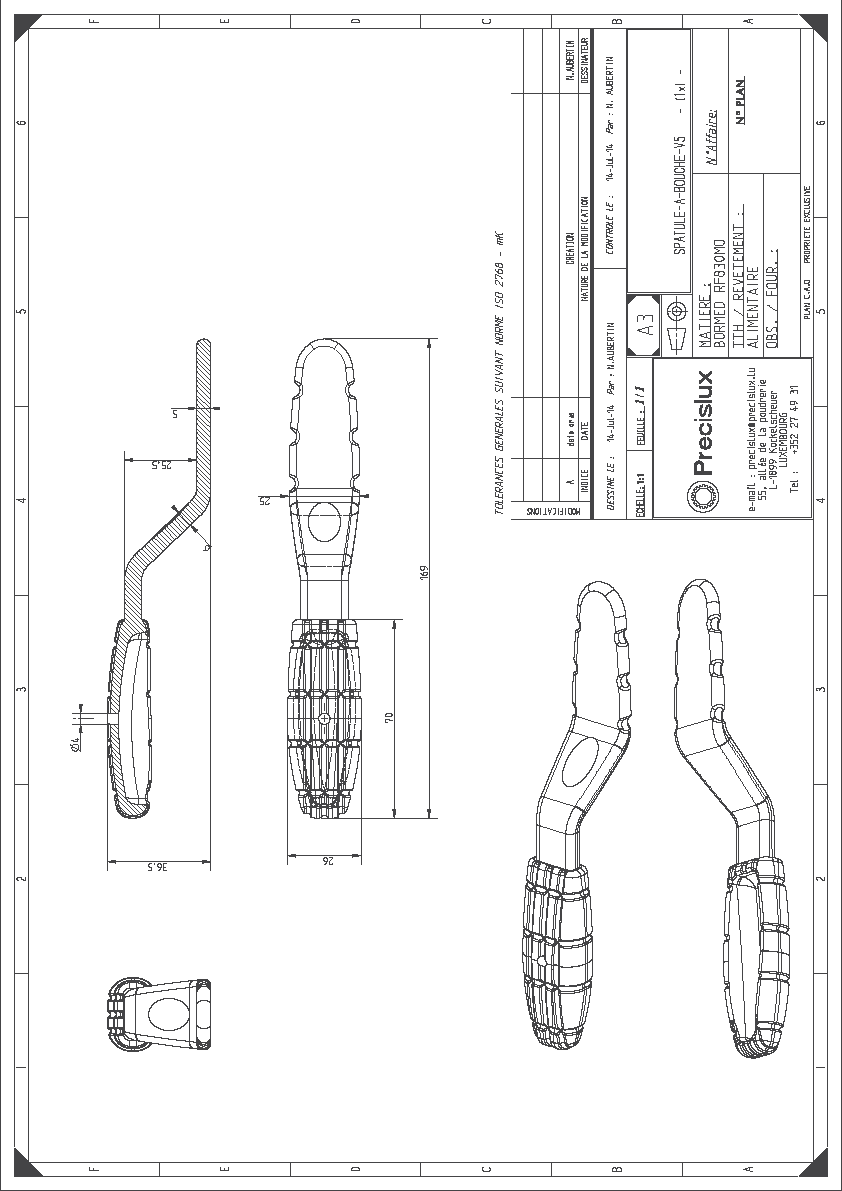


## Annex 3: Oral accessibility spatula

## illustration showing how to use it


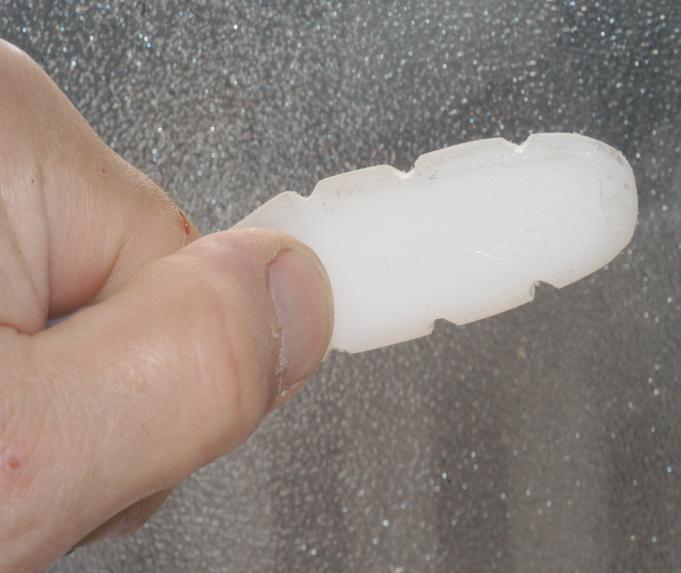


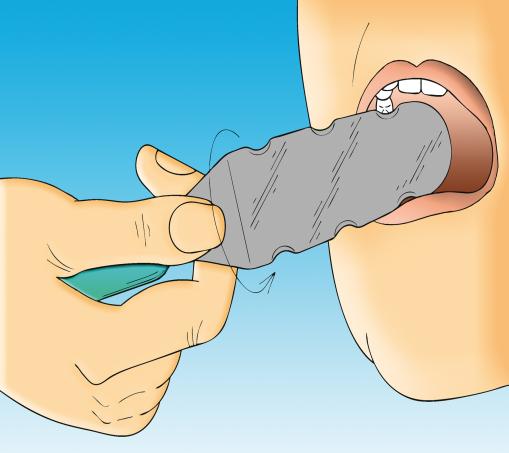


## Annex 4: operating instructions for the OAS – version N°2 of 15/01/2016

Spatula OAS

Operating instructions

1. **Description and technical characteristics.**

The OAS Spatula is a medical device that helps to attain oral accessibility. It facilitates access to the oral cavity in people who do not have the capacity to provide oral access spontaneously. It is particularly adapted to people with an impairment or dependency in whom access to the mouth is made difficult by behaviors related to their impairment or dependency.

The OAS Spatula is composed of a handle and a blade that is inserted into the mouth. The handle and the blade are interconnected by an intermediate portion that is angled to facilitate the introduction of the blade into the mouth.

- The handle is covered with retention striations that allow a good grip
- The intermediate part bears a depression that will be covered by the thumb
- The blade has a foam-like shape that is rounded at its end and bears symmetrical notches on its edges that allow the blade to wedge between the arches; this prevents slippage of the spatula inside the oral cavity.

The OAS spatula is manufactured by plastics technology. It is made from Polypropylene for medical use, which has mechanical properties that are compatible with the masticatory forces involved on the one hand and with the nature of the dental elements in contact with the OAS spatula on the other hand (it is strong enough not to break and flexible enough not to damage the hard tissue).

1. **Indications.**

The OAS Spatula is indicated in cases of oral accessibility difficulties that relate to the behavioral disorders of the person.

1. **Contraindications.**

The OAS Spatula is not indicated in cases of pathological limitation of mouth opening.

1. **Precautions for use.**

Like any restraining device, the OAS Spatula must be used with respect for the person. In particular, it must be used without applying any physical force that could traumatize the person on a physical and/or emotional level.

1. **Adverse reactions.**

The use of the OAS Spatula may potentially associate with several traumatic injuries.

These are essentially the risks of:

- mucous injuries (labial, gingival, cheek, …)
- lingual injuries
- dental fracture
- dental luxation
- dental expulsion
- nauea and vomiting
- pain
- joint complications
- inhalationor ou ingestion of dental fragments

1. **Instructions for use.**

The OAS Spatula is handled by the user in a conventional fashion. The handle is placed in the palm of the hand. The thumb is placed on the marked area on the middle part: this defines the exact position of the grip of the hand on the spatula.

Due to its thinness, the blade can be placed between the maxillary and mandibular arches at the level of the premolar-molar tooth sectors. It is introduced into the oral cavity up to the first pair of notches.

A rotational movement is then gently exerted on the handle to allow oral opening. The oral opening can be adjusted by using the other notches of the blade.

The mouth opening can then be maintained by the user, which allows constant control of the access to the oral cavity.

The contralateral zone of the oral cavity is then accessible.

Repeating this procedure on the opposite side will similarly allow access to the contralateral zone of the oral cavity.


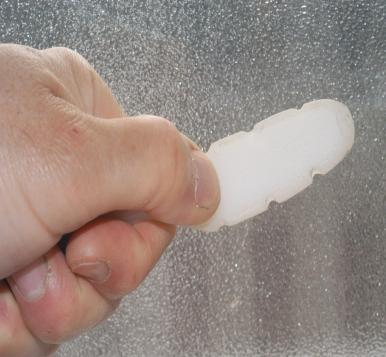

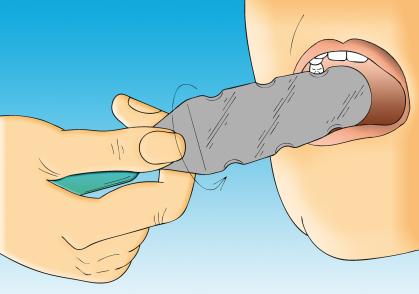


1. **Pregnancy and lactation.**

The OAS Spatula has not been tested on pregnant or lactating women.

1. **Packaging, sterilization.**

The OAS Spatula is for single use only.

It will be packaged in sterile double packaging according to a sterilization protocol ~~conventional hospital sterilization protocol (134° C to 5 bar pressure)~~ for hospital use that involves ionizing radiation with gamma rays (25 kGy). It can be stored at room temperature.

## Annex 5: data sheet for the polypropylène

##
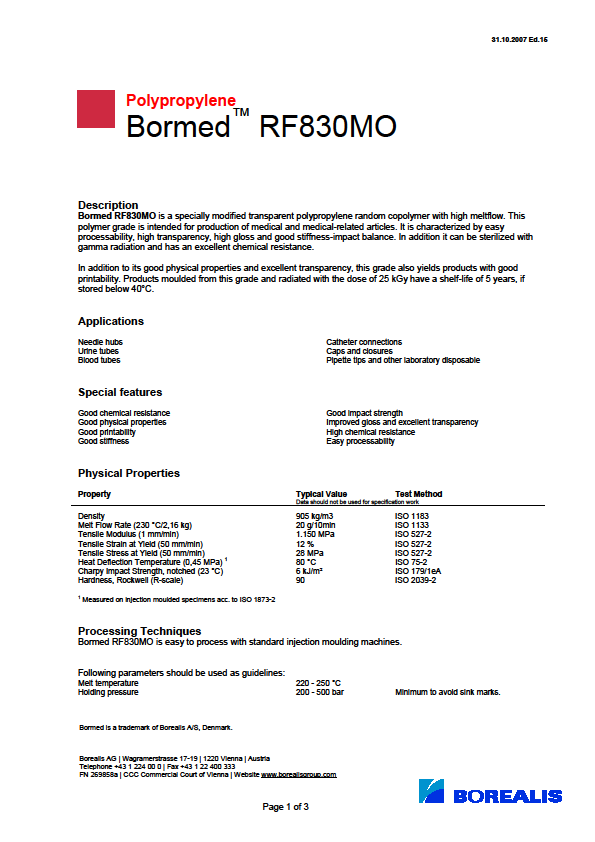


##

## Annex 6: Venham SCale

0 *Relaxed*, Relaxed, smiling, open, able to converse, best possible working conditions. Adopts the behavior desired by the dentist either spontaneously or as soon as it is requested.

1 *Uneasy*, worried. Looks directly at the operator but has a tense facial expression. Looks furtively around the environment. Sits spontaneously against the back of the chair. The hands remain down or are partially raised to signal discomfort. During a stressful maneuver, can protest briefly and quickly to indicate discomfort. The patient is willing - and able - to say what he/she feels when asked. Breath is sometimes held. Able to cooperate well with the dentist.

2 *Tense*. The tone of voice and the questions and answers indicate anxiety. Intensifies requests for information. Hands are clenched on the armrests, can reach out and be raised, but without disturbing the dentist, rests spontaneously against the chair back, but the head and neck remain tight. Accepts being held by the hand. Looks directly at the operator. During a stressful maneuver, verbal protests and discreet crying may occur. The patient interprets the situation with reasonable accuracy and continues to control his/her anxiety. The protests are more troublesome. The patient still obeys when asked to cooperate. Therapeutic continuity is preserved.

3 *Reluctant* to accept the therapeutic situation, struggles to understand the situation. Sighs often. Protests are energetic, possible crying. Lies back against the chair after several requests, the head and the neck remain tense. Slight avoidance movements. Hands clenched, gaze is sometimes fleeting. Accepts being held by the hand. Hesitates to use his/her hands to try to block the dentist's actions. Wriggles a bit. Protests verbally, weepy. Protests are not commensurate with the danger or expressed well before the danger. Copes with the situation with great reluctance. The session unfolds with difficulty.

4 *Very perturbed* by anxiety and unable to understand the situation. Significant withdrawal. Frowning, gaze is fleeting, the eyes may be intentionally closed. Vehement crying that is unrelated to the treatment. Abrupt avoidance movements. Put his/her hands on his/her mouth or on the dentist's arm but ends up letting go. Squeezes the lips, but keeps the mouth open. Frequently raises the head from the chair. Rejects body contact, but can still accept being held by the hand. Significant writhing, sometimes requiring restraint. The patient can be accessible to verbal communication and finishes the session, after a lot of effort and not without reluctance. Is trying to control him/herself. The dissociation is partial. The session is regularly interrupted by protests.

5 *Totally disconnected* from the reality of the danger, inaccessible to communication. Rejects body contact. Clamps lips and teeth. Closes the mouth and clenches the teeth as soon as possible. Shakes head violently. Cries loudly, screams, insults, struggles, is aggressive; inaccessible to verbal and non-verbal communication. Whatever the age, presents primitive reactions of flight. Attempts to escape. Restraint is required.

## Annex 7: SAE declaration form

|  | | **to be completed by the sponsor** | | | | | | | | | | | | |
| --- | --- | --- | --- | --- | --- | --- | --- | --- | --- | --- | --- | --- | --- | --- |
|  |  | **serious adverse event (SAE) NOTIFICATION sheet**  **to fax once duly completed within 24 working hours to 03 87 55 77 64** | | | | | | | | | | | | |
| **OAS: Evaluation of the effectiveness and safety of an innovative device designed to enhance the oral accessibility of people with a handicap who have behavioral disorders: the Oral Accessibility Spatula.**  **N° ID RCB (*Study registration number*): 2012-a01535-38**  **Principal investigator:** Dr. Daniel ANASTASIO  Department of Odontology, CHR METZ-THIONVILLE - Bel Air Hospital  1-3 rue du Friscaty – BP 60327 – 57126 THIONVILLE cedex  Tel: 0382558169– Email: d.anastasio@chr-metz-thionville.fr | | | | | | | | | | | First notification  Date _ _ / _ _ / _ _ _ _    Follow-up report n°: |_|  Date _ _ / _ _ / _ _ _ _ | | | |
| **Patient N° Inclusion |__|__|__| Initials: |__|-| __|**  Date of birth: _ _ / _ _ / _ _ _ _ Age (years):  Weight (kg): |__|__|__| Height (cm): |__|__|__|  Date included in the study: _ _ / _ _ / _ _ _ _ | | | | | | | | **Severity criterion**  death  life-threatening  requires hospitalization or hospitalization must be prolonged  disabled/incapacitated temporarily or permanently  birth defect, congenital malformation  other significant medical events | | | | | | |
| **Description of SAE:** | | | | | | | | | | | | | | |
| **Cause established by investigator:**  Event related to experimental medical device  Event not related to medical device  Cannot be determined  **Concomitant medications** (excluding those used to treat the SAE)  if necessary, complete with another sheet | | | | | | | | | | | | | | |
| **Name** | **Type** | | | **Route** | | **Dosage** | | | **Start date** | | | | **End date** | |
|  |  | | |  | |  | | | _ _ /_ _ /_ _ _ _ | | | | _ _ /_ _ /_ _ _ _ | |
|  |  | | |  | |  | | | _ _ /_ _ /_ _ _ _ | | | | _ _ /_ _ /_ _ _ _ | |
|  |  | | |  | |  | | | _ _ /_ _ /_ _ _ _ | | | | _ _ /_ _ /_ _ _ _ | |
|  |  | | |  | |  | | | _ _ /_ _ /_ _ _ _ | | | | _ _ /_ _ /_ _ _ _ | |
|  |  | | |  | |  | | | _ _ /_ _ /_ _ _ _ | | | | _ _ /_ _ /_ _ _ _ | |
|  |  | | |  | |  | | | _ _ /_ _ /_ _ _ _ | | | | _ _ /_ _ /_ _ _ _ | |
|  |  | | |  | |  | | | _ _ /_ _ /_ _ _ _ | | | | _ _ /_ _ /_ _ _ _ | |
| Medical and surgical history: | | | | | | | | | | | | | Dates | |
| **OAS study 2012-A01535-38** | | | | | **to be completed by the sponsor** | | | | | | | | |  |
| N° Inclusion |__|__|__| Initials: |__|-| __| | | | | | | | | | | | | | |  |
| **Description of the serious adverse event**:  Start date: _ _ / _ _ / _ _ _ _ time: |__|__| H |__|__| min  Time since the use of the experimental medical device:   hours  days  months  Description: *Predominant diagnosis or symptoms. Describe the chronology of the event and the therapeutic measures that were taken*.  **Anonymized reports of the hospitalization, examinations, and/or the laboratory results are attached:** **yes** **no** | | | | | | | | | | | | | |  |
| Action taken | | | | | | | | | | | | | |  |
| None Therapeutic action taken: | | | | | | | | | | | | | |  |
| **Evolution**  recovery without sequelae Date of recovery: _ _ / _ _ / _ _ _ _  recovery with sequelae: _ _ _ _ _ _ _ _ _ _ _ _ _ _ _ _ _  subject has not yet recovered   unknown | | | | | | | **In the case of death**: date of death: _ _ / _ _ / _ _ _ _  death unrelated to SAE  SAE may have contributed to death  death is due to SAE | | | | | | |  |
| **Name of the investigator** | | | | | **Date** | | | | | **Signature** | | | |  |
| **Section reserved for the sponsor** Date of receipt_ _ /_ _ /_ _ _ _ | | | | | | | | | | | | | |  |
|  | | | **Cause**  Event related to study  Event not related to study  Cause not determined | | | | | | | | | **SAE is**  Expected  Unexpected | |  |
| Comments: | | | | | | | | | | | | | |  |
| Request for additional information yes no | | | | | | | | | | Signature | | | |  |

**ANNEX 8: OPTIMON table**

**Definition of the risk level of the research in terms of patient safety**(based on the OPTIMON table)

| **TYPE OF STUDY** | | | | | | | |
| --- | --- | --- | --- | --- | --- | --- | --- |
|  | | | | | | | |
| **CLINICAL TRIAL OF MEDICATION, RADIOTHERAPY, GENETIC THERAPY, OR CELL THERAPY** | **SURGERY** | **MEDICAL DEVICE including IMAGING, RADIOLOGY, RADIOISTOPES** | **RISK OF THE STUDY** | | | **GENETIC PHYSIOPATHOLOGY OTHER INTERVENTIONS** | **QUALITY OF LIFE QUESTIONNAIRE PSYCHIATRY** |
| --- | Minimally invasive technique | CE-marked, class I or IIa, routine use  CE-marked, class I, off label | 🡺 | A | 🡸 | Minimally or not invasive  (including blood test)  not painful | Questionnaire without particular difficulties |
| Confirmatory study on a product with authorization or a new association that is used in accordance with the AMM (Marketing Authorization) | Technique or biopsy on an internal organ | CE-marked, class IIa, off label  CE-marked, class IIb or III, routine use  Not CE-marked, class I, not invasive and not active | 🡺 | B | 🡸 | Invasive or painful | Questionnaire affected by a serious disease |
| Confirmatory study on a product with authorization or a new association that is used diffently to the AMM (Marketing Authorization)  Exploratory study on a product with authorization or new association | Generalization of a new technique | CE-marked, class IIb, off label  CE-marked, class IIb or III, with little difference | 🡺 | C | **CONDITIONS THAT AUGMENT THE RISK**  **a) Risky INTERVENTION**, including risk of mortality or severe morbidity that is linked to the intervention, new indication, potentially dangerous withdrawal, invasive act with penetration *via* a route other than an orifcie of the body (excluding blood test)  **b) Risky INVESTIGATION**, including risk of mortality or severe morbidity that is linked to the investigation, use of a radioactive product, or little known, or without authorization, invasive act with penetration *via* a route other than an orifice of the body (excluding blood test)  **c) POPULATION AT RISk**, including risk of mortality or severe morbidity that is linked to a serious disease or to age, failure or insufficiency of a system or organ, age ≤ 2 years, age ≥ 80 years, pregnant woman, parturient woman, or lactating woman |  |  |
| Exploratory study on a product without authorization  1st study in humans (pharmacology, bioequivalence) | Development of a new technique | CE-marked, class III, off label  Not CE-marked, class I, invasive or active  Not CE-marked, class IIa or IIb or III | 🡺 | D |  |  |  |


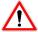
 If the OPTIMON table is used, the increased risk linked to specific populations

(Law 2004-806, cf page 4) is not counted in the LI score.

**ANNEX 9: Calculation of the "LOGISTICS, IMPACT, RESOURCES" score of the RESEARCH (A, B, C)**

**After the risk to the patient that is linked to their participation in the research is assessed, it is weighted by the impact of the research and the research strategy**.

|  | | | | | | ***Report the corresponding score*** |
| --- | --- | --- | --- | --- | --- | --- |
| **SCORE LOGISTIQUE** | **Mono/multicentric character** | **Monocentric** | | | 0 |  |
|  |  | **Multicentric** | | - Interregional - National - International | 1  2  3 | 1 |
|  |  |  |  | - 1 to 5 exterior centers - 6 to 10 exterior centers - 11 à 20 exterior centers - > 20 centers | 1  2  3  4 | 1 |
|  | **Logistical Complexity** | **Biology Circuit** | | - No - Yes | 0  1 | 0 |
|  |  | **Imaging Circuit** | | - No - Yes | 0  1 | 0 |
|  |  | **Products Circuit** | | - No - Yes | 0  1 | 1 |
|  |  | **Patient Circuit** | | - No - Yes | 0  1 | 0 |
|  |  | **Data Circuit** | | - No - Yes | 0  1 | 0 |
|  | **Number of patients expected** | | | - < 50 - 50 – 200 - > 200 | 0  1  2 | 1 |
|  | **Duration of participation per patient** | | | - < 7 days - 7 days – 6 months - 6 months – 2 years - > 2 years | 0  1  2  3 | 0 |
|  | **CRF** | | **CRF Type** | - e-CRF - Paper | 0  1 | 1 |
|  |  |  | **Number of pages or variables** | - < 20 pages or < 200 variables - 20 to 50 pages or 200 to 500 - > 50 pages or > 500 | 0  1  2 | 0 |
|  | **LOGISTICAL SCORE** | | | | | **5 / 20** |
| **SCORE IMPACT** | **Study Design** | **Randomized** | | - No - Yes | 0  1 | 1 |
|  |  | **Blinded Research** | | - No - Yes | 0  1 | 0 |
|  | **Weakness of the data** | **Rare pathology** | | - No - Yes | 0  1 | 0 |
|  |  | **Pediatric, special population*** | | - No - Yes | 0  1 | 1 |
|  |  | **Seasonal malady** | | - No - Yes | 0  1 | 0 |
|  |  | **Emergency/Resuscitation Situation** | | - No - Yes | 0  1 | 0 |
|  | **Collection of sensitive data** | | | - No - Yes | 0  1 | 0 |
|  | **Impact of the results** | | | - Other/Publication - Medico-eco / STIC - Made at the request of the authorities | 0  1  2 | 0 |
|  | **Impact/risk relating to the media and/or politics** | | | - No - Yes | 0  1 | 0 |
|  | **Potential patent** | | | - No - Yes | 0  1 | 1 |
|  | **Study can be integrated into an AMM (marketing authorization) file (or extension)** | | | - No - Yes | 0  1 | 0 |
|  | **DGOS, ANR, Europe, Inca Financing** | | | - No - Yes | 0  1 | 0 |
|  | **Industrial or other partnership contract** | | | - No - Yes | 0  1 | 0 |
|  | **IMPACT SCORE** | | | | | **3 / 14** |
| **SCORE RESSOURCES** | **Experience of the coordinating investigator** | | | - Experience as coordinator - Experience as principal investigator (not coordinator) - Neither as coordonnator nor as principal investigator | 0  1  2 | **2** |
|  | **Experience of the associated investigative centers** | | | - > 50% of the centers - < 50% of the centers | 0  1 | **1** |
|  | **Presence of research staff in the coordinating center** | | | - Experience in study coordination - Experience in study investigation - No or without experience | 0  1  2 | **0** |
|  | **Presence of dedicated research staff in associated centers** | | | - > 50% of the centers - < 50% of the centers | 0  1 | **1** |
|  | **RESOURCES SCORE** | | | | | **4 / 6** |
| **TOTAL LOGISTIC + IMPACT + RESOURCES SCORE** | | | | | | **12/ 40** |

**Definition of the level of monitoring according to patient risk and the "LIR" score**

| **Risk to patient** | **total "Logistic Impact Resources” score** | |
| --- | --- | --- |
|  | **1 to 19** | **>20** |
| **A** | Minimal level | Intermediate level |
| **B** | Minimal level | Intermediate level |
| **C** | Intermediate level | Elevated level |
| **D** | Elevated level | |
