# Supplementary material for: Efficacy, tolerability, and safety of an innovative medical device for improving oral accessibility during oral examination in special-needs patients: A multicentric clinical trial
Source: PLoS One. 2020 Sep 28;15(9):e0239898. doi: 10.1371/journal.pone.0239898 (PMC7521731; doi:10.1371/journal.pone.0239898)
Supplement: S3 File — (DOC) [file pone.0239898.s005.doc]

**Protocole de Recherche Clinique**

Avis favorable du CPP Est III le 07 /07 /2015

Autorisation de l’ANSM le 28/11/2014

Version N°4.0 du 11/08/2017

Modifiant substantiellement la version N°3.0 du 07/09/2016

N° IDRCB : 2012-A01535-38

**EVALUATION DE L’EFFICACITE ET DE LA SECURITE D’EMPLOI D’UN DISPOSITIF INNOVANT PERMETTANT D’AMELIORER L’ACCESSIBILITE BUCCALE CHEZ DES PERSONNES EN SITUATION DE HANDICAP PRESENTANT DES TROUBLES DU COMPORTEMENT :**

**LA SPATULE D’ACCESSIBILITE BUCCALE (SAB)**

**Promoteur**

CHR de METZ-THIONVILLE

1 allée du château

CS45001

57085 Metz Cedex 03

**Investigateur Coordonnateur**

Dr Daniel ANASTASIO

Service d’Odontologie

CHR METZ-THIONVILLE – Hôpital Bel Air

1-3, rue du Friscaty – BP 60327 – 57 126 THIONVILLE Cedex

Tel : 03 82 55 81 69 / Mail : d.anastasio@chr-metz-thionville.fr

**PRINCIPAUX CORRESPONDANTS**

**Investigateur Coordonnateur**

Dr Daniel ANASTASIO

Service d’Odontologie

CHR METZ-THIONVILLE – Hôpital Bel Air

1-3, rue du Friscaty – BP 60327 – 57 126 THIONVILLE Cedex

Tel : 03 82 55 81 69 / Mail : d.anastasio@chr-metz-thionville.fr

**Investigateurs associés**

**Méthodologiste**

Nom : Christophe GOETZ

Adresse : **CHR METZ THIONVILLE**

**Hôpital Mercy**

**1 allée du château**

**CS 45001**

**57085 Metz cedex 03**

Tél : 03 87 55 37 46

Mail : c.goetz@chr-metz-thionville.fr

**Chef de projet**

Nom : Nadia OUAMARA

Adresse : **CHR METZ THIONVILLE**

**Hôpital Mercy**

**1 allée du château**

**CS 45001**

**57085 Metz cedex 03**

Tél : 03 87 55 77 52

Mail : [n.ouamara@chr-metz-thionville.fr](mailto:n.ouamara@chr-metz-thionville.fr)

# Historique des mises à jour du protocole

| Version | Date | Raison de la mise à jour |
| --- | --- | --- |
| V 1.0 | 17/11/2014 | Première version |
| V 1.1 | 01/06/2015 | - Changement dans les contacts Promoteur : Chef de projet et Attaché de recherche clinique - Suppression de l’annexe 10 : la grille budgétaire - Ajout de deux centres investigateurs associés supplémentaires : - CHR Metz Thionville - Hôpital de Mercy - Centre de Santé Dentaire - Fondation Sonnenhof à Bischwiller - Mise à jour du calendrier prévisionnel de l’étude. |
| V 2.0 | 15/01/2016 | Modification substantielle (MS1) : changement du mode de stérilisation |
| V 3.0 | 07/09/2016 | Modification substantielle (MS2) :   - Modification de l’investigateur du centre investigateur CHU de Dijon par Dr Ludwig LOISON-ROBERT   Modification substantielle (MS3) :   - Ajout d’un objectif secondaire : confort de l’opérateur, dont le critère de jugement secondaire est le score à l’échelle numérique. |
| V 4.0 | 11/08/2017 | Modification substantielle (MS4) :   - Ajout d’un nouveau centre investigateur associé : Centre Hospitalier Emile Durkheim-Epinal et d’un nouvel investigateur principal : Dr Amélie DALSTEIN - Mise à jour du calendrier prévisionnel de l’étude : prolongation de la durée d’inclusion |

**SOMMAIRE**

**1. RESUME DU PROTOCOLE**

**2. Justification scientifique et description générale de la recherche**

**3. Objectifs de la recherche**

**4. Conception de la recherche**

**5. Sélection et exclusion des personnes de la recherche**

**6. DISPOSITIF MEDICAL**

**7. EVALUATION DE L’EFFICACITE**

**8. Evaluation de la sécurité**

**9. Statistiques**

**10. Droit d'accès aux données et documents source**

**11. Contrôle et assurance de la qualité**

**12. Considérations éthiques ET REGLEMENTAIRES**

**13. Traitement des données et conservation des documents et des données relatives à la recherche**

**14. Financement et assurance**

**15. Règles relatives à la publication**

**16. Liste des annexes**

**17. REFERENCES BIBLIOGRAPHIQUES**

**1. RESUME DU PROTOCOLE**

| Titre | *SAB : Evaluation de l’efficacité et de la sécurité d’emploi d’un dispositif médical innovant permettant d’améliorer l’accessibilité buccale chez des personnes en situation de handicap présentant des troubles du comportement :la Spatule d’Accessibilité Buccale (SAB)* |
| --- | --- |
| Promoteur | *CHR METZ THIONVILLE* |
| Investigateur Coordinateur | *Dr Daniel ANASTASIO*  *Service d’Odontologie*  *CHR METZ-THIONVILLE – Hôpital Bel Air*  *1-3, rue du Friscaty – BP 60327 – 57 126 THIONVILLE Cedex*  *Tel : 03 82 55 81 69 / Mail : d.anastasio@chr-metz-thionville.fr* |
| Version du protocole | *V4.0 du 11/08/2017* |
| Justification/ contexte | *Chez les personnes en situation de handicap comportemental, l’accessibilité de la cavité buccale est parfois limitée voire impossible. Ces patients expriment des réflexes de défense en limitant spontanément l’amplitude de l’ouverture buccale ou en ne maintenant cette dernière que durant des périodes très courtes.*  *Les moyens actuels de maintien de la cavité buccale ouverte (cales) sont souvent inadaptés à ces patients et aucun dispositif alternatif n’est décrit dans la littérature.*  *Face à cette problématique, la réalisation des examens bucco-dentaires de prévention ou les soins apportés à ces patients sont difficiles.* |
| Objectif Principal | *Evaluation de l’accessibilité buccale lors d’un examen bucco-dentaire initial sans puis avec utilisation de la spatule d’accessibilité buccale (SAB).* |
| Objectifs Secondaires | *Evaluation de l’innocuité de la SAB*  *Evaluation de la tolérance de l’examen sans puis avec la SAB*  *Evaluation du confort de l’opérateur de la spatule SAB* |
| Critère de Jugement Principal | *Visibilité et examen à la sonde des secteurs dentés incisivo-canins, prémolaires et molaires évalués par un score d’accessibilité buccale établi en fonction des zones intra buccales accessibles sans et avec la SAB (score de 0=absence d’accessibilité à 12=accessibilité totale)* |
| Critères de Jugement Secondaires | *Recensement des effets indésirables et de leur fréquence respective.*  *Echelle comportementale de Venham*  *Echelle numérique de satisfaction pour l’opérateur* |
| Méthodologie / Schéma de l’etude | *Etude interventionnelle prospective, multicentrique, en ouvert*  *Une seule visite est prévue par patient.* |
| Critères d’Inclusion des Sujets | - *Patient mineur ou majeur présentant une déficience accompagnée d’une difficulté comportementale avérée ou exprimée lors de l’examen bucco-dentaire* - *Score de 2 ou plus sur l’échelle de Venham, lors d’une approche graduée vers la cavité buccale de la personne (toucher de la main, toucher labial, essai d’écartement labial)* - *Information spécifique à l’étude délivrée aux titulaires de l’autorité parentale pour les mineurs, au représentant légal pour les mineurs et majeurs sous tutelle, au patient et au curateur pour les majeurs sous curatelle et formulaire de consentement éclairé signé* |
| Critères de Non-Inclusion des Sujets | - *Femme enceinte ou allaitante* - *Absence de couverture sociale* - *Patient sous sauvegarde de justice* |
| Procédures | *Tout patient présentant un handicap comportemental et consultant pour un bilan de prévention ou pour un soin bucco-dentaire est examiné par un opérateur d’inclusion qui valide les critères d’inclusion / non inclusion, et en particulier, le comportement par l’échelle de Venham (seuls les patients avec un score de 2 ou plus, c’est-à-dire opposants, lors d’une approche vers la cavité buccale sont inclus).*  *Pour chaque patient éligible, un score d’accessibilité buccale (noté de 0/absence d’accessibilité à 12/accessibilité totale) est établi, d’abord sans la SAB (examen standard), puis avec la SAB.*  *Les incidents et/ou blessures éventuelles sont également recueillis selon une grille standardisée.*  *L’échelle de Venham est de nouveau mesurée lors de l’examen bucco-dentaire sans et avec spatule.*  *A la fin de la visite, l’opérateur évalue sa satisfaction envers l’utilisation de la SAB par une échelle numérique (score de 0 : peu satisfaisant à 10 : très satisfaisant).* |
| Nombre de Patients | *Sans SAB, la proportion de patients dont le score d’accessibilité buccale atteint au moins le niveau 8 est de 20%.*  *Le protocole fait l’hypothèse que l’utilisation de la SAB permet d’améliorer de 20% cette proportion (soit 40% des patients atteignant au moins le score 8). Un effectif total de 140 patients est nécessaire pour valider cette hypothèse, avec un risque α de 5% et une puissance de 90%. Cet effectif est porté à 200 patients afin de prendre en compte un potentiel effet centre et un potentiel effet investigateur, ainsi qu’un taux de sortie d’étude de 10%.* |
| Durée de la Recherche | *Une seule visite est prévue par patient.*  *La durée totale de l’étude correspond donc à la période de recrutement, soit 33 mois.*  *Début prévu de la recherche : Mars 2016*  *Fin du recrutement : Décembre 2018*  *Fin de l’étude : Juin 2019* |
| analyse statistique | *L’analyse principale sera la comparaison des scores d’accessibilité buccale lors de l’examen avec et sans la SAB. Chaque sujet sera son propre témoin et les examens seront toujours réalisés d’abord sans la SAB puis avec la SAB par le même chirurgien dentiste* |
| Retombées attendues | *Le résultat attendu est la démonstration d’une meilleure qualité d’examen sans risque pour le patient. La principale perspective est la mise à disposition de la SAB, aux chirurgiens-dentistes pour améliorer les indications thérapeutiques et la qualité des interventions, puis aux parents, auxiliaires de vie, personnel soignant afin de rendre possible ou de faciliter la mise en œuvre d’une hygiène bucco-dentaire adaptée chez des personnes en situation de handicap ou de dépendance.*  ***Les Bénéfices attendus sont donc les suivants :***   - *dépistage précoce des pathologies bucco-dentaires* - *efficacité accrue des soins réalisés* - *limitation des indications de soins sous anesthésie générale* - *aide au brossage afin d’augmenter significativement le niveau d’hygiène bucco-dentaire de ces personnes*   *- prévention accrue par la mise à disposition de cette spatule aux parents, auxiliaires de vie, accompagnants et professionnels de la santé.* |

**Liste des abréviations**

| ANSM | Agence Nationale de Sécurité du Médicament et des Produits de Santé |
| --- | --- |
| ARC | Attaché de Recherche Clinique |
| BPC | Bonnes Pratiques Cliniques |
| CPP | Comité de Protection des Personnes |
| CSP | Code de la Santé Publique |
| CNIL | Commission Nationale de l’Informatique et des Libertés |
| CRF | Case Report Form (cahier d’observation) |
| EIG | Evènement Indésirable Grave |
| EIGI | Effet Indésirable Grave Inattendu |
| ICH | International Conference on Harmonization (Conférence internationale pour l'harmonisation) |
| MR | Méthodologie de Référence |
| SAB | Spatule d’Accessibilité Buccale |
| SUSAR | Suspected Unexpected Serious Adverse Reaction |
| TEC | Technicien d'Etude Clinique |
|  |  |

**2. Justification scientifique et description générale de la recherche**

**2.1. Dénomination et description du dispositif médical de l’essai**

La présente recherche porte sur une spatule d’accessibilité buccale dénommée « SAB ».

Un dispositif d’aide à l’accessibilité buccale peut être défini selon les critères suivants :

- aide efficace à l’ouverture buccale
- facilité de mise en œuvre
- maintient « à distance » de l’ouverture buccale par l’équipe soignante
- aide à l’examen de la cavité buccale, au brossage et aux soins dentaires éventuels.

La SAB est un dispositif médical **innovant** d’aide à l’accessibilité buccale.

La réflexion globale est la création d’un outil facilitant l’accessibilité buccale chez des personnes en situation de handicap ou de dépendance pour qui l’accès en bouche est difficile du fait d’une réaction de protection lors de l’examen bucco-dentaire. Cet outil s’adresse particulièrement aux personnes présentant un trouble du comportement lié à la déficience.

La SAB répond aux critères suivants :

- forme adaptée à la cavité buccale des adultes comme des enfants
- non traumatique pour les structures dentaires
- non traumatique pour les tissus muqueux
- facile d’utilisation
- bonne préhension
- hygiénique, décontaminable et stérilisable selon les standards hospitaliers (une seule stérilisation avant utilisation, la spatule étant à usage unique)

Il s’agit d’un dispositif d’aide technique à une prise en charge préventive ou curative et non d’un dispositif thérapeutique.

Par ailleurs, ce dispositif n’est pas adapté à la prise en charge de personnes présentant une limitation pathologique de l’ouverture buccale.

**2.2. Résumé des résultats des essais non cliniques et des essais cliniques disponibles et pertinent au regard de la recherche concernée**

Il n’existe pas actuellement de dispositif médical de ce type.

L’originalité de cette spatule réside dans la possibilité d’accéder à l’intérieur de la cavité buccale et ensuite de la maintenir ouverte durant l’examen.

Ce dispositif est plus adapté qu’une cale intra-buccale classique, plus difficile à mettre en place et à maintenir entre les arcades dentaires durant l’examen.

Les dispositifs actuels d’aide à l’accessibilité buccale sont les suivants :

- ouvre-bouches métalliques
- cales intra-buccales en caoutchouc
- dispositifs en forme de toupie

Aucun de ces instruments ne répond de manière satisfaisante aux critères d’accessibilité buccale.

Du fait de son caractère innovant, la SAB a fait l’objet d’un dossier de demande de brevet en date du 29/03/2010. Le brevet d’invention a été délivré le 08/11/2013.

**2.3. Résumé des bénéfices et des risques prévisibles et connus pour les personnes se prettant à la recherche**

**Bénéfices pour le patient :**

- meilleure accessibilité de la cavité buccale
- possibilité de dépistage bucco-dentaire de lésions carieuses et parodontales
- lutte contre la douleur éventuelle en cas de lésions avérées
- évaluation du niveau d’hygiène de la personne examinée
- diagnostic bucco-dentaire
- possibilité d’établir un plan de traitement adapté à la nature de la déficience et au degré de coopération

**Effets indésirables :**

- possibilité de lésions buccales (voir chap 4.4.2).
- inhalation ou ingestion de dent ou fragment dentaire
- pas d’autres risques induits par cette recherche pour les personnes incluses dans le protocole.

Une évaluation de la sécurité d’emploi du dispositif sera faite lors de l’étude

**2.4. Description et justification des modalites d’utilisation et duree de traitement**

La problématique de la prise en charge de la personne présentant une déficience comportementale se situe dans la réaction instinctive de protection.

La cavité buccale est une zone particulière, carrefour aérodigestif, siège de l’alimentation, de la respiration et de l’expression de l’oralité.

La protection s’exprime par un mouvement de repli avec fléchissement de la tête en avant et fermeture buccale.

L’intérêt de l’utilisation de la SAB réside dans la possibilité de lever la barrière de protection de fermeture par une introduction buccale facilitée et performante.

La notion de durée de traitement n’est pas concernée par l’étude qui repose sur l’évaluation de l’efficacité du dispositif lors d’un examen bucco-dentaire simple.

**2.5. Déclaration indiquant que la recherche sera conduite conformement au protocole, aux BPC et aux dispositions législatives réglementaires en vigueur**

La recherche sera conduite conformément au protocole, aux Bonnes Pratiques Cliniques et à la règlementation en vigueur.

**2.6. Description de la population à étudier**

La population étudiée dans le cadre de cette étude est constituée de patients majeurs et/ou mineurs présentant un handicap comportemental et qui sont :

- soit adressés au service d’Odontologie pour un bilan de prévention ou pour un soin bucco-dentaire (quel qu’il soit)
- soit pris en charge dans le cadre d’une visite annuelle de dépistage bucco-dentaire au sein de l’établissement spécialisé dont dépend le patient

La population concernée par cette étude est donc constituée de personnes particulièrement protégées dans le cadre de la recherche biomédicale (articles L1121-8 et L1121-7 du Code de la Santé Publique), à savoir des patients majeurs faisant l’objet d’une mesure de protection légale ainsi que de patients mineurs.

**3. Objectifs de la recherche**

**3.1. Objectif Principal**

L’objectif principal de notre étude est l’évaluation de l’accessibilité buccale lors d’un examen bucco-dentaire initial, sans puis avec utilisation de la SAB.

**3.2. Objectifs secondaires**

Les objectifs secondaires sont :

- l’évaluation de l’innocuité de la spatule
- l’évaluation de la tolérance de l’examen, sans puis avec la spatule.
- L’évaluation du confort de l’opérateur

**3.3. Objectifs de toute etude ancillaire eventuelle**

**Non applicable**

**4. Conception de la recherche**

**4.1. critères d’évaluation**

- *Critère d’évaluation principal*

Le critère principal de jugement est la visibilité et l’examen à la sonde des secteurs dentés incisivo-canins, prémolaires et molaires. Ce critère inclut un abord vestibulaire et palatin/lingual.

La visibilité et l’examen à la sonde sont cotés et permettent le calcul d’un « score d’accessibilité buccale » situé entre 0 (absence d’accessibilité) et 12 (accessibilité totale). La construction de ce score est décrite au paragraphe 4.4.1.

- *Critères d’évaluation secondaires*

L’innocuité de la spatule sera évaluée à l’aide d’un recensement des effets indésirables et de leurs fréquences respectives.

La tolérance de l’examen, sans puis avec la SAB, sera évaluée à l’aide de l’échelle comportementale de Venham (échelle validée).

Le confort de l’opérateur dans l’utilisation de la spatule SAB sera évalué à la fin de chaque visite par l’échelle numérique de satisfaction (score de 0 : peu satisfaisant à 10 : très satisfaisant), par l’opérateur utilisant la SAB.

## 4.2. méthodologie de la recherche

Le recrutement des patients se fera :

- soit dans le service d’Odontologie, dans le cadre d’un bilan de prévention ou pour un soin bucco-dentaire (quel qu’il soit)
- soit dans l’établissement spécialisé dont dépend le patient, dans le cadre d’une visite annuelle de dépistage bucco-dentaire.

Pour l’ensemble des patients, l’évaluation se déroulera en une seule visite.

La population concernée par cette étude est constituée de personnes particulièrement protégées dans le cadre de la recherche biomédicale, à savoir des patients majeurs faisant l’objet d’une mesure de protection légale ainsi que des patients mineurs.

Les patients reçoivent donc une information adaptée à leur capacité de compréhension de la part de l’investigateur.

L’information est également délivrée :

- aux titulaires de l’autorité parentale pour les mineurs
- au représentant légal pour les mineurs et majeurs sous tutelle
- au curateur pour les majeurs sous curatelle

Le formulaire de consentement éclairé sera signé :

- par les titulaires de l’autorité parentale pour les mineurs
- par le représentant légal pour les mineurs et majeurs sous tutelle
- par la personne qui se prête à la recherche assistée de son curateur pour les majeurs sous curatelle

L’adhésion personnelle des patients sera toujours recherchée, **dans la mesure du possible**, et il ne pourra être passé outre à leur refus.

Une fois le formulaire de consentement signé, l’investigateur évalue les critères d’inclusion définitifs du patient : calcul du score obtenu sur l’échelle de Venham, lors d’une approche graduée vers la cavité buccale de la personne (toucher de la main, toucher labial, essai d’écartement labial). Le patient est inclus dans l’étude si le score de Venham est égal ou supérieur à 2, c'est-à-dire qu’il y a une opposition à l’examen.

Si le patient répond à l’ensemble des critères d’inclusion et ne présente aucun critère de non inclusion, l’investigateur procède au premier examen de la cavité buccale **sans** la SAB.

Il consigne les paramètres d’évaluation de l’accessibilité et les incidents éventuels (voir paragraphe 4.4)

Il calcule également le score obtenu à l’échelle de Venham pendant l’examen sans SAB (évaluation de la tolérance en cours d’examen).

Dans un second temps, l’investigateur procède à l’examen de la cavité buccale **avec** la SAB.

Il consigne les paramètres d’évaluation de l’accessibilité et les incidents éventuels (voir paragraphe 4.4).

Il calcule également le score obtenu à l’échelle de Venham pendant l’examen avec SAB (évaluation de la tolérance en cours d’examen).

Enfin, après avoir réalisé les deux examens (sans puis avec la SAB), l’opérateur évalue son confort d’utilisation de la spatule SAB par rapport à l’examen habituel sans SAB.

## 4.3. Description des mesures prises pour réduire et éviter les biais

Chaque sujet sera son propre témoin.

Les examens seront toujours réalisés d’abord sans la SAB puis avec la SAB car il n’est pas souhaitable de réaliser un examen en débutant par une approche avec un dispositif contraignant. L’examen sans la SAB puis avec la SAB sera réalisé par le même chirurgien dentiste pour maximiser le confort du patient (ne pas lui imposer deux intervenants différents et un temps d’examen allongé si changement d’intervenant).

Ce plan d’analyse permet de neutraliser l’effet opérateur, mais peut introduire un éventuel biais d’évaluation car le même chirurgien dentiste procède aux 2 évaluations. Cependant le seul plan d’analyse qui permettrait de s’en libérer serait un essai en groupes parallèles dont l’un des groupes ne serait examiné qu’avec la SAB, sans approche préalable, ce qui n’est pas souhaitable pour le patient. Afin de limiter au maximum ce biais d’évaluation, l’accessibilité buccale sera évaluée avec un score objectif consistant en un dénombrement des secteurs visibles de la cavité buccale (voir 4.4.1), et tous les investigateurs seront sensibilisés sur l’importance du respect strict des consignes d’utilisation de ce score afin d’éviter un biais d’évaluation.

L’examen avec SAB succédera toujours à un examen sans SAB, mais il n’y a pas lieu de parler de biais d’accoutumance en raison de cet ordre imposé, car la SAB ne doit, dans tous les cas, pas être utilisée d’emblée dans un examen de la cavité buccale

Il est précisé que l’investigateur coordonnateur de cette étude qui est aussi l’inventeur de la SAB, pourra être opérateur d’inclusion, mais ne pourra pas réaliser les examens de la cavité buccale et l’évaluation de l’accessibilité buccale, ceci afin d’éviter tout conflit d’intérêt.

Cette étude sera réalisée en ouvert car l’utilisation du dispositif à l’étude ne permet pas de garder le patient et/ou l’investigateur en aveugle.

## 4.4. dipsositif medical à l’étude

## 4.4.1 Paramètres d’évaluation de l’accessibilité buccale

Les paramètres suivants seront évalués sans puis avec la SAB :

- visibilité intra-buccale des secteurs incisivo-canins maxillaires et mandibulaires droits et gauches
- visibilité intra-buccale des secteurs prémolaires maxillaires et mandibulaires droits et gauches
- visibilité intra-buccale des secteurs molaires maxillaires et mandibulaires droits et gauches
- examen à la sonde des secteurs incisivo-canins maxillaires et mandibulaires droits et gauches
- examen à la sonde des secteurs prémolaires maxillaires et mandibulaires droits et gauches
- examen à la sonde des secteurs molaires maxillaires et mandibulaires droits et gauches

Chaque item est noté de 0 à 2 :

- 0 : résultat négatif à droite et à gauche
- 1 : résultat positif sur l’un des deux côtés examinés
- 2 : résultat positif sur les deux côtés examinés

L’examen bucco-dentaire sans utilisation de la SAB sera côté de 0 à 12.

L’examen bucco-dentaire avec utilisation de la SAB sera côté de 0 à 12.

## 4.4.2 Paramètres d’évaluation des incidents liés à l’utilisation de la SAB

11 items sont retenus pour évaluer la sécurité d’utilisation de la SAB. Ces incidents sont couramment décrits dans l’utilisation des cales intrabuccales, ouvre-bouches métalliques ou toupies:

- blessure labiale
- blessure gingivale
- blessure jugale
- blessure linguale
- fracture dentaire
- luxation dentaire
- expulsion dentaire
- réflexes nauséeux, vomissements
- douleurs
- complications articulaires
- autres

Chaque item sera noté 1 (oui) ou 0 (non).

## 4.5. Durée prévue de participation des personnes et description de la chronologie et de la durée de toutes les périodes de l'essai

Durée de participation pour un patient : 1 visite (environ 30 minutes)

Durée de recrutement des patients : 33 mois

Durée totale de la recherche : 39 mois

Durée totale prévisionnelle de la recherche (durée d'inclusion et durée de participation) : 39 mois

Le patient ne participe qu’à une seule visite :

- signature du consentement par le représentant du patient s’il est mineur ou majeur sous tutelle, par le patient lui-même s’il est majeur sous curatelle

- vérification des critères d’inclusion et de non inclusion

Examen de la cavité buccale **sans** la SAB:

- évaluation de l’accessibilité et les incidents éventuels (voir paragraphe 4.4)

- score obtenu à l’échelle de Venham pendant l’examen sans SAB.

Examen de la cavité buccale **avec** la SAB :

- évaluation de l’accessibilité et les incidents éventuels (voir paragraphe 4.4).

- score obtenu à l’échelle de Venham pendant l’examen avec SAB.

L’opérateur finit chaque visite par l’évaluation du confort d’utilisation de la spatule SAB en utilisant l’échelle numérique.

Il n’y a pas de suivi spécifique à l’étude à l’issue de cette visite.

**4.6. Dispositions mises en oeuvre en vue du maintien de l'insu et procédures de levée de l'insu, le cas échéant.**

Non applicable, étude réalisée en ouvert

**4.7. Identification de toutes les données à recueillir directement dans les cahiers d'observation, qui seront considérées comme des données source.**

Non applicable

**4.8. collection d’échantillons biologiques**

Non applicable

**5. Sélection et exclusion des personnes de la recherche**

La population concernée par cette étude est constituée de personnes particulièrement protégées dans le cadre de la recherche biomédicale, à savoir des patients majeurs faisant l’objet d’une mesure de protection légale ainsi que de patients mineurs.

La recherche sur ces populations se justifie car :

- elle répond aux besoins et priorités de santé de ces populations

- les résultats de la recherche seront bénéfiques pour ces populations

- l’étude ne peut être réalisée sur une autre population

- les risques et inconvénients sont mineurs au regard des bénéfices attendus.

Par ailleurs, il est nécessaire de tester l’efficacité et l’innocuité de la spatule sur des mineurs afin de confirmer que la SAB est adaptée à des cavités buccales de petite taille.

**5.1. Critères d'inclusion**

- Patient mineur ou majeur présentant une déficience accompagnée d’une difficulté comportementale avérée ou exprimée lors de l’examen bucco-dentaire
- Score de 2 ou plus sur l’échelle de Venham, lors d’une approche graduée vers la cavité buccale de la personne (toucher de la main, toucher labial, essai d’écartement labial)
- Information spécifique à l’étude délivrée aux titulaires de l’autorité parentale pour les mineurs, au représentant légal pour les mineurs et majeurs sous tutelle, au patient et au curateur pour les majeurs sous curatelle et formulaire de consentement éclairé signé

**5.2. Critères de non-inclusion**

- Femme enceinte ou allaitante
- Absence de couverture sociale
- Patient sous sauvegarde de justice

**5.3. Procédure d'arrêt prématuré de la recherche ou d'exclusion (ARRÊT DU TRAITEMENT + ARRÊT DU SUIVI)**

**5.3.1. Critères et modalités d’arrêt de traitement / Dm et exclusion**

- *Critères d’arrêt prématuré*

Impossibilité de réaliser les évaluations, sans puis avec spatule, par un comportement mettant en jeu l’intégrité physique de la personne (score 5 sur l’échelle de Venham).

- *Les modalités de suivi des personnes exclues de l’étude*

Les patients présentant un score 5 sur l’échelle de Venham seront examinés en dehors de l’étude à l’aide des techniques médicales suivantes :

- sous sédation par inhalation d’un mélange équimolaire d’oxygène et de protoxyde d’azote
- sous sédation par utilisation de Midazolam (intra-rectal ou intra-veineux)
- Sous anesthésie générale si nécessaire.

**5.3.2. Modalités des recueil des données et de suivis**

Les données seront recueillies dans un cahier d’observation papier, à partir du dossier source du patient.

Les patients ne participent qu’à une seule visite dans le cadre de l’étude. Il n’y a pas de suivi prévu.

**5.3.3. Modalités de remplacement des personnes**

Le nombre de sujets nécessaire intègre un taux d’échec de l’examen de 10%. Les patients sortant d’étude ne seront donc pas remplacés.

**5.3.4 Modalités de suivi des personnes**

Non applicable.

**5.4. Modalités de recrutement**

Les patients seront recrutés :

- soit au service d’Odontologie pour un bilan de prévention ou pour un soin bucco-dentaire (quel qu’il soit)
- soit au sein de l’établissement spécialisé dont dépend le patient, dans le cadre d’une visite annuelle de dépistage bucco-dentaire

La période d’inclusion sera de 33 mois.

**6. DISPOSITIF MEDICAL**

**6.1. Dispositif médical utilise**

*Figure 1 : photo de la Spatule d’Accessibilité Buccal SAB*


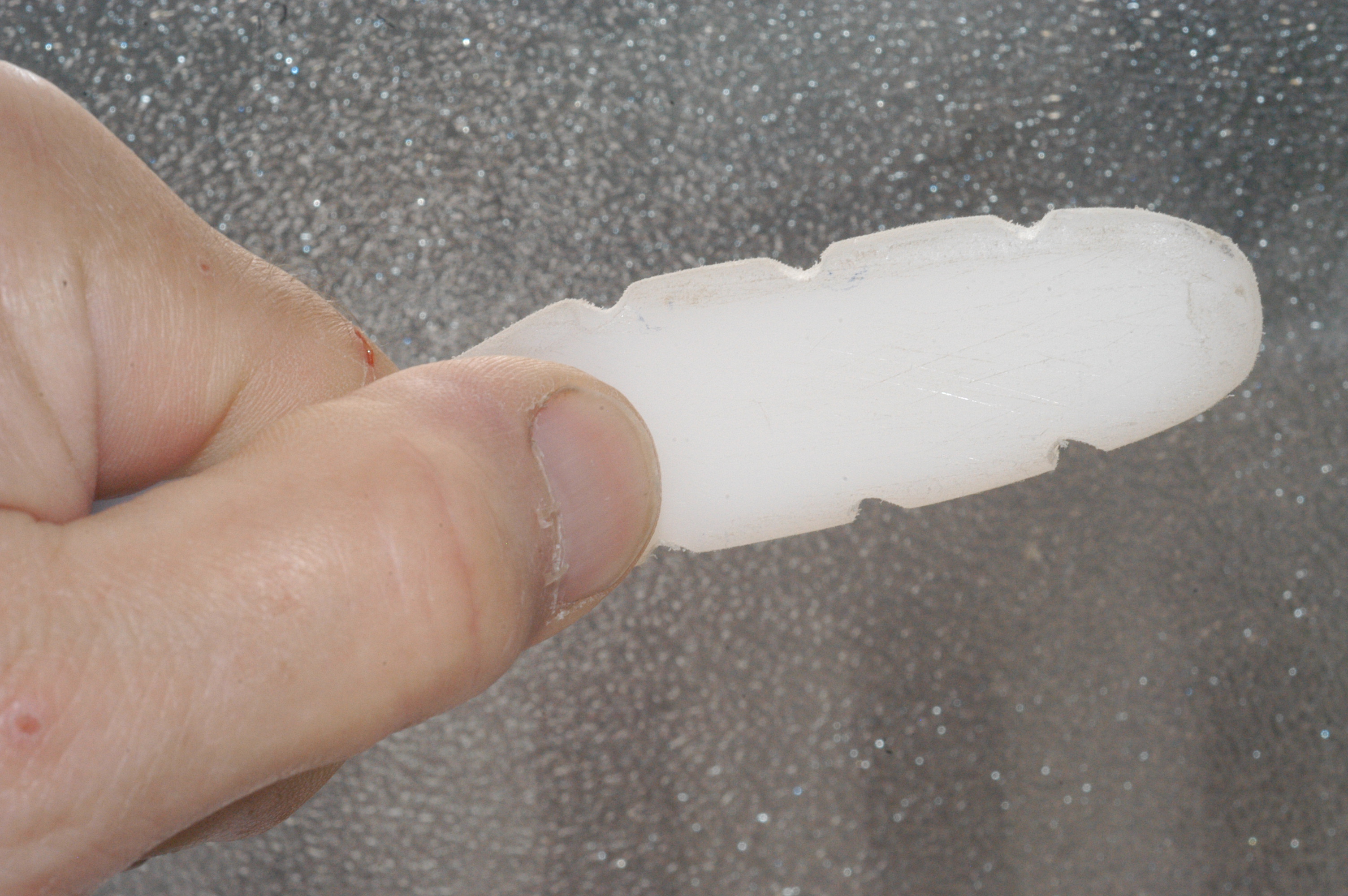


## Modalités d’utilisation de la SAB.

La SAB est constituée d’une lame et d’un manche de préhension (voir annexes 1, 2 et 3). La lame permet, de par sa forme, d’être insérée dans la cavité buccale entre les arcades dentaires.

Un mouvement de rotation du manche permet d’exercer une force suffisante pour permettre l’ouverture buccale.

La lame est pourvue d’encoches afin de caler les dents maxillaires et mandibulaires et créer ainsi un calage des arcades lors de l’examen buccal.

La spatule est entièrement en polypropylène transparent Bormed™ RF830MO pour ne pas traumatiser les éléments dentaires en cas de morsure de la spatule.

La forme de la lame est particulièrement étudiée afin de ne pas léser les différents tissus de la cavité buccale.

La matière est compatible avec une stérilisation aux normes actuelles hospitalières : une seule stérilisation par radiations ionisantes aux rayons gamma (25 kGy).

Le polypropylène Bormed™ RF830MO peut être stérilisé par rayonnement gamma avec une dose de 25kGy. Ce mode de stérilisation permet une période de stérilité de 5 ans pour un stockage à température ambiante. (Cf fiche technique du produit annexe 5)

Les utilisateurs de cette spatule lors de l’essai clinique sont des chirurgiens-dentistes habitués à l’accès buccal.

L’utilisation ne demande pas, à priori, de formation de la part des investigateurs. Cependant, une notice d’utilisation a été réalisée (annexe 4) afin de normaliser la technique de mise en œuvre du dispositif médical. Une attention particulière sera demandée aux utilisateurs sur l’aspect d’aide à l’accessibilité buccale en respectant l’intégrité physique de la personne.

Les SAB, lors de l’essai clinique, seront stérilisées selon les normes hospitalières en vigueur et conditionnées en double emballage stérile individuel. Elles proviennent toutes du même lot de fabrication afin d’être rigoureusement identiques. Chaque spatule sera à usage unique puis détruite comme tout déchet médical.

**6.2. Description de tout medicament, dm (autres que ceux expérimentaux) utilise pour les besoins de la recherche**

Non applicable

**6.3 Médicament et traitements autorises et interdit pendant la recherche**

- Aucun dispositif médical d’aide à l’ouverture buccale (autre que la SAB) ne sera utilisé durant l’étude.
- Aucune médication sédative ne sera utilisée en dehors des traitements en cours.

**6.4. Méthode de suivi de l’observance**

La SAB n’est pas un dispositif thérapeutique mais un dispositif d’aide technique à une prise en charge préventive ou curative. Chaque patient ne participe qu’à une seule visite. Il n’y a donc pas de suivi de l’observance.

**7. EVALUATION DE L’EFFICACITE**

## 7.1. Description des paramètres d'évaluation de l'efficacité

Les données recueillies dans le cadre de l’évaluation de l’efficacité de la SAB sont décrites au paragraphe 4.4.1

## ***7.2. Méthodes et calendrier prévus pour mesurer, recueillir et analyser les paramètres d'évaluation de*** l'efficacité.

Les méthodes et le calendrier prévus pour mesurer, recueillir et analyser ces paramètres, sont décrits au paragraphe 4.5.

Pour l’ensemble des patients, l’évaluation de l’efficacité sera mesurée lors de l’unique visite réalisée dans le cadre de l’étude.

**8. Evaluation de la sécurité :**

**8.1. Description des paramètres d’évaluation de la sécurité**

Les données recueillies dans le cadre de la surveillance du patient sont décrites au paragraphe 4.4.2.

**8.2. Méthodes et calendrier prévus pour mesurer, recueillir et analyser les paramètres d’évaluation de la sécurité**

Chaque patient est évalué une seule fois dans le cadre de cette étude. Les paramètres d’évaluation de la sécurité seront recensés au cours de cette visite unique.

**8.3 Procédures mises en place en vue de l’enregistrement et de la notification des évènements indésirables**

**8.3.1 Définitions**

Un **événement indésirable** est une manifestation nocive survenant chez une personne qui se prête à une recherche biomédicale, que cette manifestation soit liée ou non à la recherche ou au dispositif sur lequel porte cette recherche.

Un **effet indésirable** d’un dispositif médical est toute réaction nocive et non désirée à un dispositif médical ou tout incident qui aurait pu entraîner cette réaction si une action appropriée n'avait pas été effectuée, chez une personne qui se prête à la recherche ou chez l'utilisateur du dispositif médical ou tout effet lié à une défaillance et néfaste pour la santé d'une personne qui se prête à la recherche.

**Un évènement indésirable grave ou effet indésirable grave (EIG)** est un évènement ou un effet ayant pu contribuer au décès du patient, à la mise en jeu de son pronostic vital, à son hospitalisation ou à la prolongation de son hospitalisation, à une incapacité ou à un handicap important ou durable, ou se traduire par une anomalie ou une malformation congénitale ou tout événement jugé significatif par l’investigateur.

**La liste des effets indésirables attendus** liés à l’étude est établie à partir du document de référence.

Un EIG est inattendu (EIGI) lorsqu’il ne figure pas sur cette liste.

**Un fait nouveau** peut être : fréquence inattendue d’un EIG attendu, événement indésirable grave lié à la procédure de l’essai, efficacité insuffisante dans les maladies à pronostic vital, données non cliniques.

**8.3.2 Document de référence permettant de définir le caractère attendu d’un EIG**

Il n’existe pas de document de référence pour le dispositif médical à l’étude, puisqu’il s’agit d’un dispositif médical innovant.

Une brochure pour l’investigateur a été réalisée par le Dr Anastasio, concepteur de la spatule.

Les évènements attendus sont les mêmes que ceux fréquemment rencontrés lors de l’utilisation des cales intra-buccales en caoutchouc, des ouvre-bouches métalliques ou des dispositifs en forme de toupie.

**8.3.3 Liste des effets indésirables attendus**

Aucun effet indésirable n’est actuellement connu dans le cadre de l’utilisation du dispositif médical à l’étude, puisqu’il s’agit d’un dispositif médical innovant.

Les évènements indésirables attendus sont ceux retenus pour l’évaluation des incidents liés à la spatule :

- blessure labiale
- blessure gingivale
- blessure jugale
- blessure linguale
- fracture dentaire
- luxation dentaire
- expulsion dentaire
- réflexes nauséeux, vomissements
- douleurs
- complications articulaires

Les effets indésirables graves attendus sont l’inhalation (par fausse route) ou l’ingestion d’une dent ou d’un fragment dentaire et qui nécessiteraient une hospitalisation.

L’inhalation ou l’ingestion d’une dent ou d’un fragment de dent peut se produire suite à une luxation ou une fracture dentaire, évènements survenant sur une dent qui bougeait préalablement à la réalisation de l’examen.

On attend au maximum 1 à 2 EIG liés à la SAB sur la totalité des patients inclus dans l’étude.

**8.3.4 Transmission des EIG et faits nouveaux**

Dès qu’un investigateur prend connaissance d’un EIG ou d’un fait nouveau, il le notifie sans délai au promoteur en faxant la fiche de déclaration d’EIG (annexe 7).

- S’il s’agit d’un effet indésirable grave inattendu (EIGI), le promoteur entre en contact avec l’investigateur pour rédiger un rapport initial qui sera transmis à l’ANSM, au CPP et à l’investigateur coordonnateur dans les 7 jours en cas de décès ou de mise en jeu du pronostic vital, sinon dans les 15 jours.

Lorsque l’évènement n’est pas résolu à la date d’envoi du fax, l’investigateur est tenu d’envoyer un rapport complémentaire (dans les 8 jours en cas de décès ou de mise en jeu du pronostic vital, sinon dans les 15 jours) afin de documenter l’évolution ou de réactualiser les données manquantes.

- S’il s’agit d’un EIG lié à la procédure de mise en œuvre du dispositif médical expérimental, la déclaration se fait à l’ANSM selon les mêmes modalités que précédemment et au CPP tous les trimestres.
- S’il s’agit de fait nouveau, le promoteur le déclarera à l’ANSM et au CPP dans les 15 jours maximum et les informations complémentaires pourront être apportées sous 15 jours supplémentaires.
- S’il s’agit d’un effet indésirable grave attendu, il sera colligé par le promoteur en vue de la rédaction des rapports annuels de sécurité.

**8.3.5 Transmission des effets indésirables non graves**

Ils seront décrits succinctement par l’investigateur dans le cahier d’observation à la section dédiée aux évènements indésirables.

**8.4. Modalités et durée de suivi des personnes suite à la survenue d’évènements indésirables**

Lorsque un évènement indésirable grave persiste, y compris après la fin de l’étude, l’investigateur suivra le patient jusqu’à ce que l’évènement soit considéré comme résolu et transmettra les données de suivi au promoteur**.**

**8.5. Comités spécifiques de la recherche (variable selon les protocoles)**

**8.5.1 Comité de pilotage**

Il sera constitué de l’initiateur clinicien du projet, du biostatisticien en charge du projet et des représentants du promoteur.

Il définira l'organisation générale et le déroulement de la recherche et coordonnera les informations.

Il déterminera initialement la méthodologie et décidera en cours de recherche des conduites à tenir dans les cas imprévus, surveillera le déroulement de la recherche en particulier sur le plan de la tolérance et des évènements indésirables.

### 8.5.2 Comité de surveillance indépendant

La nature de cette étude ne justifie pas la mise en place d’un comité de surveillance indépendant.

### 8.5.3 Comité Indépendant d'Evaluation des Evénements Critiques

La nature de cette étude ne justifie pas la mise en place d’un comité indépendant d’évaluation des évènements critiques.

**8.6 Rapports de sécurité**

- Rapports annuels de sécurité : le promoteur rédige les rapports annuels de sécurité et les transmet à l’ANSM, au CPP et à l’investigateur coordonnateur. L’investigateur coordonnateur transmettra au promoteur toutes les données nécessaires à la rédaction de ce rapport.
- Rapport final : il est rédigé par le promoteur et l’investigateur coordonnateur dans un délai d’un an après la fin de l’étude. Tous les investigateurs sont informés des résultats de l’étude. Un résumé est adressé à l’ANSM par le promoteur.

**9. Statistiques**

**9.1. description des méthodes statistiques prévues, y compris du calendrier des analyses intermédiaires prévues**

L’analyse principale consistera à

1. Décrire l’échantillon pour apprécier la nécessité et la faisabilité d’utilisation de la SAB,
2. Vérifier les critères d’inclusion et la représentativité de l’échantillon final,
3. Comparer les pourcentages de patients ayant un score d’accessibilité buccale supérieur ou égal à 8 selon l’utilisation ou non de la SAB, à l’aide d’un test de MacNemar. Les moyennes des scores seront également comparées selon un test de Student pour séries appariées. Il sera pour ces analyses recherché un éventuel effet centre et un éventuel effet investigateur.
4. Evaluer l’innocuité de la SAB en quantifiant et en décrivant les effets indésirables observés.

**9.2. nombre prévu de personnes à inclure dans la recherche, avec sa justification statistique**

Sans SAB, la proportion de patients dont le score d’accessibilité buccale atteint au moins le niveau 8 est de 20%.

Le protocole fait l’hypothèse que l’utilisation de la SAB permet d’améliorer de 20% cette proportion (soit 40% des patients atteignant au moins le score 8). Un effectif total de 120 patients est nécessaire pour valider cette hypothèse, avec un risque α de 5% et une puissance de 90%. Cet effectif est porté à 200 patients afin de prendre en compte un potentiel effet centre et un potentiel effet investigateur, ainsi qu’un taux de sortie d’étude de 10%.

**Une étude de faisabilité** a été menée dans le service d’Odontologie du CHR METZ-THIONVILLE sur une période de 12 mois (janvier à décembre 2009). Au cours de cette période, 104 patients répondant aux critères d’inclusion de notre étude ont été pris en charge par le service d’Odontologie, ce qui confirme la faisabilité des objectifs de recrutement fixés.

**9.3. degré de signification statistique prévu**

Le risque α est fixé à 5%

**9.4. critères statistiques d'arrêt de la recherche**

Aucun.

**9.5. méthode de prise en compte des données manquantes, inutilisées ou non valides**

Les sujets sans données complètes ne seront pas utilisés pour l’analyse comparative des scores d’accessibilité buccale. Ils seront décrits et comparés aux sujets à données complètes.

**9.6. gestion des modifications apportées au plan d'analyse de la stratégie initiale**

Aucune gestion particulière n’est prévue a priori.

Toute modification doit faire l’objet d’une discussion explicite entre l’investigateur et le méthodologiste.

**9.7. choix des personnes à inclure dans les analyses.**

Les sujets sans données complètes ne seront pas utilisés pour l’analyse comparative des scores d’accessibilité buccale. Ils seront décrits et comparés aux sujets à données complètes.

**10. Droit d'accès aux données et documents source**

Toutes les données et informations concernant le patient resteront strictement confidentielles. Les personnes ayant un accès direct conformément aux dispositions législatives et réglementaires en vigueur, notamment les articles L.1121-3 et R.5121-13 du code de la santé publique (par exemple, les investigateurs, les personnes chargées du contrôle de qualité, les moniteurs, les assistants de recherche clinique, les auditeurs et toutes personnes appelées à collaborer aux essais) prennent toutes les précautions nécessaires en vue d'assurer la confidentialité des informations relatives aux médicaments expérimentaux, aux essais, aux personnes qui s'y prêtent et notamment en ce qui concerne leur identité ainsi qu’aux résultats obtenus. Les données collectées par ces personnes au cours des contrôles de qualité ou des audits sont alors rendues anonymes.

**11. Contrôle et assurance de la qualité**

**11.1 Monitoring**

Le niveau de risque attribué à l’étude défini à partir de la grille OPTIMON (annexe 8) est B (dispositif de classe I non marqué CE). Selon le score Logistique et Impact-Ressources du sous-groupe de travail « Qualité de la promotion » des DRCI (annexe 9), un monitoring de niveau MINIMAL sera nécessaire.

L’ARC représentant du promoteur effectuera des visites du centre investigateur au rythme du monitoring de niveau MINIMAL :

- Visite d’ouverture du centre : avant la 1^ère^inclusion, pour une mise en place du protocole et prise de connaissance avec les différents intervenants de la recherche biomédicale.

- Lors des visites suivantes, seuls les consentements seront monitorés. Si les consentements sont non-conformes, les dossiers des patients seront monitorés de façon aléatoire sur le site.

Les feuillets des cahiers d’observation seront déliassés et récupérés par l’ARC.

L'investigateur principal ainsi que les autres investigateurs qui incluent ou assurent le suivi des personnes participant à la recherche s’engagent à recevoir l’ARC à intervalles réguliers.

Lors de ces visites sur site et en accord avec les Bonnes Pratiques Cliniques, les éléments suivants seront revus :

- Respect du protocole et des procédures définies pour la recherche,
- Examen des documents sources : notification de la signature du consentement du patient ou de la signature du titulaire de l’autorité parentale ou du tuteur dans le dossier patient.

- Visite de fermeture : récupération des derniers feuillets déliassés des cahiers d’observation, bilan à la pharmacie, archivage des documents de la recherche biomédicale.

A ce titre, l’investigateur s’engage à mettre à la disposition de l’ARC lors de ses visites de monitoring :

⮚ Les dossiers médicaux des patients

⮚ Les cahiers de recueil de données

⮚ Les formulaires de consentement des patients inclus.

### 11.2 Transcription des données dans le cahier d’observation

Toutes les informations requises par le protocole doivent être fournies dans le cahier d’observation et une explication donnée par l’investigateur pour chaque donnée manquante.

Les données devront être transférées dans les cahiers d'observation au fur et à mesure qu'elles sont obtenues, qu'il s'agisse de données cliniques ou para-cliniques. Les données devront être copiées de façon nette et lisible à l'encre noire dans ces cahiers (ceci afin de faciliter la duplication et la saisie informatique).

Les données erronées dépistées sur les cahiers d'observation seront clairement barrées et les nouvelles données seront copiées sur le cahier avec les initiales et la date par le membre de l'équipe de l'investigateur qui aura fait la correction.

L'anonymat des sujets sera assuré par un numéro de code et les initiales de la personne qui se prête à la recherche sur tous les documents nécessaires à la recherche, ou par effacement par les moyens appropriés des données nominatives sur les copies des documents source, destinés à la documentation de la recherche.

Les données informatisées sur un fichier seront déclarées à la CNIL selon la procédure adaptée au cas.

**12. Considérations éthiques ET REGLEMENTAIRES**

Le promoteur et les investigateurs s’engagent à ce que cette recherche soit réalisée en conformité avec la loi n°2004-806 du 9 août 2004, ainsi qu’en accord avec les Bonnes Pratiques Cliniques (I.C.H. version 4 du 1^er^ mai 1996 et décision du 24 novembre 2006) et la déclaration d’Helsinki (Principes éthiques applicables aux recherches médicales sur des sujets humains, Séoul 2008).

La recherche est conduite conformément au présent protocole. Les investigateurs s’engagent à respecter le protocole en tous points en particulier en ce qui concerne le recueil du consentement et la notification et le suivi des événements indésirables graves.

Chaque investigateur s'engagera à respecter les obligations de la loi et à mener la recherche selon les B.P.C., en respectant les termes de la déclaration d'Helsinki en vigueur.

12.1 Demande d’autorisation auprès de l’Ansm

Pour pouvoir démarrer la recherche, le promoteur doit soumettre un dossier de demande d’autorisation auprès de l'autorité compétente l’ANSM. L'autorité compétente, définie à l'article L. 1123-12, se prononce au regard de la sécurité des personnes qui se prêtent à une recherche biomédicale, en considérant notamment la sécurité et la qualité des produits utilisés au cours de la recherche conformément, le cas échéant, aux référentiels en vigueur, leur condition d'utilisation et la sécurité des personnes au regard des actes pratiqués et des méthodes utilisées ainsi que les modalités prévues pour le suivi des personnes.

12.2 Demande d’avis au Comité de Protection des Personnes

En accord avec l'article L.1123-6 du Code de Santé Publique, le protocole de recherche doit être soumis par le promoteur à un Comité de Protection des Personnes. L'avis de ce comité est notifié à l’autorité compétente par le promoteur avant le démarrage de la recherche.

12.3 Modifications

Les modifications devront être qualifiées en substantielles ou non.

Une modification substantielle est une modification susceptible, d'une manière ou d'une autre, de modifier les garanties apportées aux personnes qui se prêtent à la recherche biomédicale (modification d’un critère d’inclusion, prolongation d’une durée d’inclusion, participation de nouveaux centres,…).

Après le commencement de la recherche, toute modification substantielle de celle-ci à l’initiative du promoteur doit obtenir, préalablement à sa mise en oeuvre, un avis favorable du comité et une autorisation de l’autorité compétente. Dans ce cas, si cela est nécessaire, le comité s’assure qu’un nouveau consentement des personnes participant à la recherche est bien recueilli.

Toute modification substantielle devra faire l’objet par le promoteur d’une demande d’autorisation auprès de l’ANSM et/ou d’une demande d’avis du CPP.

12.4 Déclaration CNIL

Cette recherche entre dans le cadre de la « Méthodologie de référence » (MR-001) en application des dispositions de l’article 54 alinéa 5 de la loi du 6 janvier 1978 modifiée relative à l’informatique, aux fichiers et aux libertés. Ce changement a été homologué par décision du 5 janvier 2006. Le CHR de Metz-Thionville a signé un engagement de conformité à cette « Méthodologie de référence ».

Seules les données nécessaires à la recherche seront recueillies. Le patient a néanmoins le droit de s’opposer à ce que les données le concernant fassent l’objet d’un traitement automatisé. Le patient aura à tout moment le droit d’accéder aux données le concernant. Il aura également le droit de demander à ce que les données inexactes ou devenues inexactes soient rectifiées. Il pourra à tout moment exercer ses droits auprès du Dr ANASTASIO, investigateur coordonnateur.

Pour toutes les informations de nature médicale, ses droits pourront être exercés directement ou par l’intermédiaire du médecin de son choix.

Dans le cas où le consentement a été donné par la famille (ou la personne de confiance), seul le patient est en mesure d’accéder aux données le concernant tel que décrit précédemment.

**12.5 Note d’information et Consentement éclairé**

La population concernée par cette étude est constituée de personnes particulièrement protégées dans le cadre de la recherche biomédicale, à savoir des patients majeurs faisant l’objet d’une mesure de protection légale (tutelle ou curatelle) ainsi que de patients mineurs.

- **Pour les patients majeurs sous tutelle et les patients mineurs :**

Avant de recueillir le consentement du représentant légal ou des titulaires de l’autorité parentale du patient, l’investigateur s’engage à délivrer au patient une information claire, loyale et la plus adaptée et complète possible sur l'étude envisagée ; il lui remet également la notice d’information adaptée à ses capacités de compréhension.

L’information est également délivrée :

- aux titulaires de l’autorité parentale pour les mineurs
- au représentant légal pour les mineurs et majeurs sous tutelle

L’information porte sur :

- les objectifs et les contraintes de l'étude pour le patient
- sur le droit, pour le représentant légal, de refuser que le patient participe à l'étude
- sur le droit, pour le représentant légal, de faire quitter l’étude au patient à tout moment

Lorsque l'essentiel de l'information aura été donné au patient et au représentant légal du patient et lorsque l'investigateur se sera assuré que ce dernier a bien compris les implications de la participation du patient à l'essai, le consentement écrit du représentant légal sera recueilli par l’investigateur. L’adhésion du patient sera toujours recherchée.

Le formulaire de consentement sera signé en deux exemplaires originaux par les titulaires de l’autorité parentale ou le tuteur et le médecin investigateur :

⮚ Un exemplaire sera remis aux titulaires de l’autorité parentale ou au tuteur suivant les cas

⮚ Un exemplaire sera conservé et archivé par l'investigateur.

- **Pour les patients majeurs sous curatelle** :

Le patient, assisté de son curateur, recevra une information orale et écrite, claire, loyale, complète et adaptée à ses capacités de compréhension.

Après avoir disposé d’un temps de réflexion et avoir eu la possibilité de poser toutes les questions qu’il souhaite, s’il est d’accord pour participer à l’étude, le patient, assisté de son curateur, pourra signer lui-même son consentement, en deux exemplaires originaux. Un exemplaire sera remis au patient et le 2^ème^ sera conservé et archivé par l’investigateur.

### 12.6 Rapport final de la recherche

Le rapport final de la recherche sera écrit en collaboration par le coordonnateur et le biostatisticien pour cette recherche. Ce rapport sera soumis à chacun des investigateurs pour avis. Une fois qu'un consensus aura été obtenu, la version finale devra être avalisée par la signature de chacun des investigateurs et adressée au promoteur dans les meilleurs délais après la fin effective de la recherche. Un rapport rédigé selon le plan de référence de l’autorité compétente doit être transmis à l’autorité compétente ainsi qu’au CPP dans un délai de un an, après la fin de la recherche, s’entendant comme la dernière visite de suivi du dernier sujet inclus. Ce délai est rapporté à 90 jours en cas d’arrêt prématuré de la recherche.

13. Traitement des données et conservation des documents et des données relatives à la recherche

Les documents d’une recherche entrant dans le cadre de la loi sur les recherches biomédicales doivent être archivés par toutes les parties pendant une durée de 15 ans après la fin de la recherche*.*

cet archivage indexé comporte :

- Les copies de courrier d’autorisation de l’ANSM et de l’avis obligatoire du CPP
- Les versions successives du protocole (identifiées par le n° de version et la date de version),
- Les courriers de correspondance avec le promoteur,
- Les consentements signés des sujets sous pli cacheté (dans le cas de sujets mineurs signés par les titulaires de l’autorité parentale, dans le cas de patient sous tutelle par le tuteur) avec la liste ou registre d’inclusion en correspondance,
- Le cahier d’observation complété et validé de chaque sujet inclus,
- Toutes les annexes spécifiques à l’étude,
- Le rapport final de l’étude provenant de l’analyse statistique et du contrôle qualité de l’étude (double transmis au promoteur).
- Les certificats d’audits éventuels réalisés au cours de la recherche

La base de données ayant donné lieu à l’analyse statistique doit aussi faire l’objet d’archivage par le responsable de l’analyse (support papier ou informatique).

**14. Financement et assurance**

**14.1 Assurance**

Le promoteur a souscrit auprès de SHAM pour toute la durée de l'étude une assurance garantissant sa propre responsabilité civile ainsi que celle de tout intervenant impliqué dans la réalisation de l'étude, indépendamment de la nature des liens existant entre les intervenants et le promoteur.

### financement

Un 1^er^ financement a été obtenu en 2008 : il s’agit d’un soutien de 3000€ versé par la Mutuelle Nationale des Hospitaliers suite au 11^ème^ trophée « Innovation Handicap » remporté par le service d’odontologie de l’hôpital Bel Air.

Des financements complémentaires ont été obtenus par le CHR Metz Thionville pour la réalisation de l’étude dans le cadre du PHRC-I 2011.

Les spatules SAB seront fabriquées par la société PrecisLux.

500 spatules seront fournies, toutes issues du même lot.

**15. Règles relatives à la publication**

Le CHR de Metz-Thionville est propriétaire des données et aucune utilisation ou transmission à un tiers ne peut être effectuée sans son accord préalable.

Le CHR de Metz-Thionville doit être mentionné comme étant le promoteur de la recherche biomédicale et comme soutien financier le cas échéant.

**16. Liste des annexes**

Annexe 1 : Spatule d’Accessibilité Buccale : illustration

Annexe 2 : Schéma de la Spatule d’Accessibilité Buccale

Annexe 3 : Spatule d’Accessibilité Buccale : Illustration des modalités d’utilisation

Annexe 4 : Notice d’utilisation de la Spatule d’Accessibilité Buccale

Annexe 5 : Fiche technique du polypropylène Bormed™ RF830MO

Annexe 6 : Echelle de Venham

Annexe 7 : Fiche de notification des EIG

Annexe 8 : Grille OPTIMON

Annexe 9 : Score LIR.

##

**17. REFERENCES BIBLIOGRAPHIQUES**

**Collado V, Faulks D, Hennequin M**. A survey of the difficulties encountered during routine hygiene and health care by persons with special needs. *Disabil Rehabil. 2008; 30(14):1047-54*.

**Davis MJ**. Issues in access to oral heath care for special care patients. *Dent Clin North Am. 2009; 53(2):169-81.*

**Glassman P**. A review of guidelines for sedation, anaesthesia, and alternative interventions for people with special needs. *Spec Care Dentist. 2009; 29(1):9-16*.

**Christensen GJ**. Special oral hygiene and preventive care for special needs. *J Am Assoc. 136;8:1141-1143.*

**Hennequin M, Moysan V, Jourdan D, Dorin M, Nicolas E**. Inequalities in oral heath for health for children with disabilities : a french national survey in special schools. *PLoS One. 2008;3(6):e2564.*

**De Jongh A, Van Houtem C, Van der Schoof M, Resida G, Broers D**. Oral health status, treatment needs, and obstacles to dental care among noninstitutionalized children with severe mental disabilities in the Netherlands. *Special Care Dentist. 2008;28(3):111-5*.

**Marchall J, Sheller B, William BJ, Mancl L, Cowan C**. Cooperation predictors for dental patients with autism. *Pediatr Dent. 2007;29(5):369-376*.

**Nussbaum BL**. Dental care for patients who are unable to open their mouths. *Dent Clin North Am. 2009;53(2):323-8.*

**Romer M**. Consent, restraint, and people with special needs : a review. *Spec Care Dentist. 2009 ;29(1) :58-66.*

**Annexe 1 : Spatule d’Accessibilité Buccale (SAB)**

**illustration**


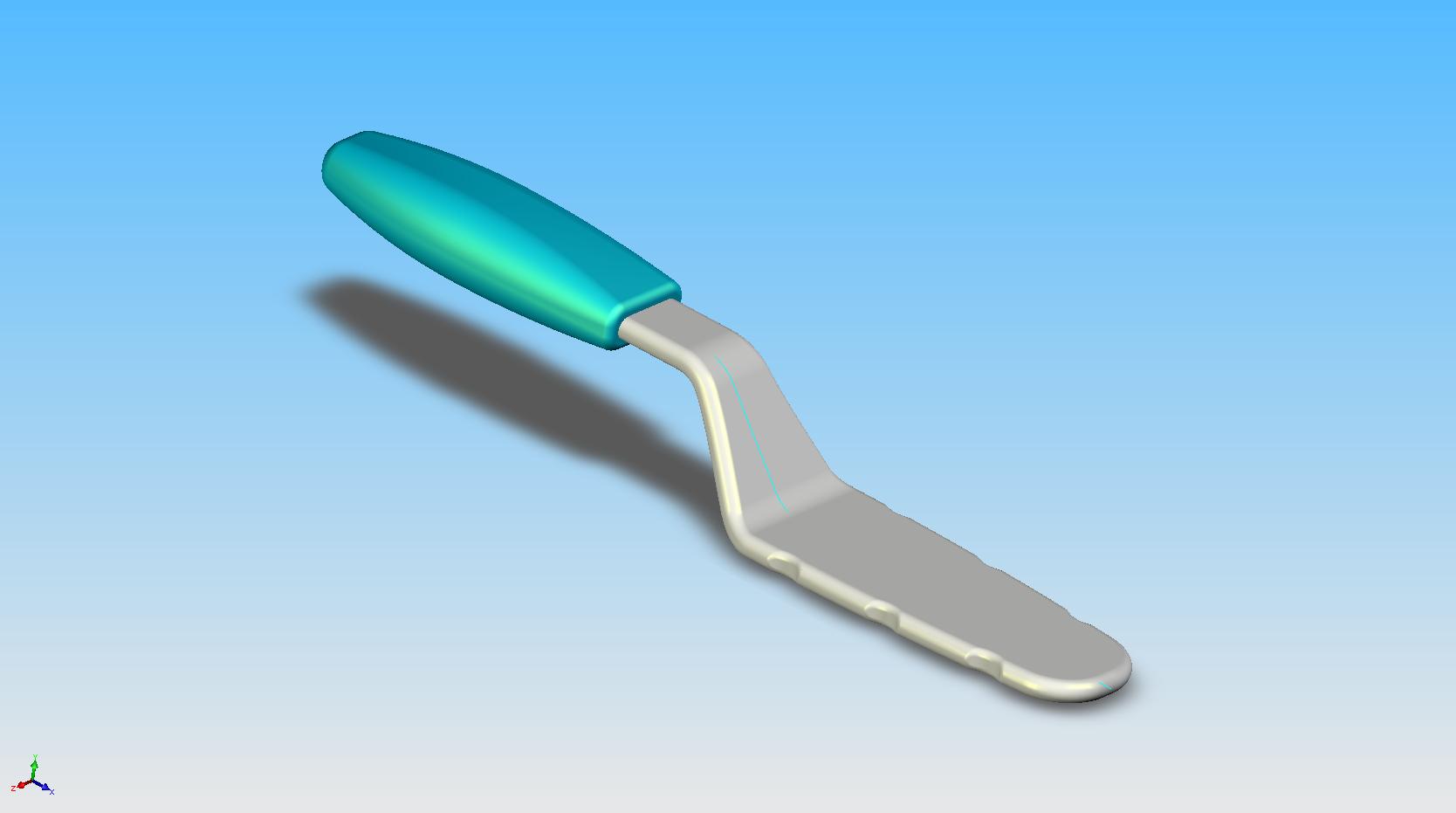


**Annexe 2: schéma de la spatule d’accessibilité buccale**

## Annexe 3: Spatule d’Accessibilité Buccale

## illustration des modalités d’utilisation


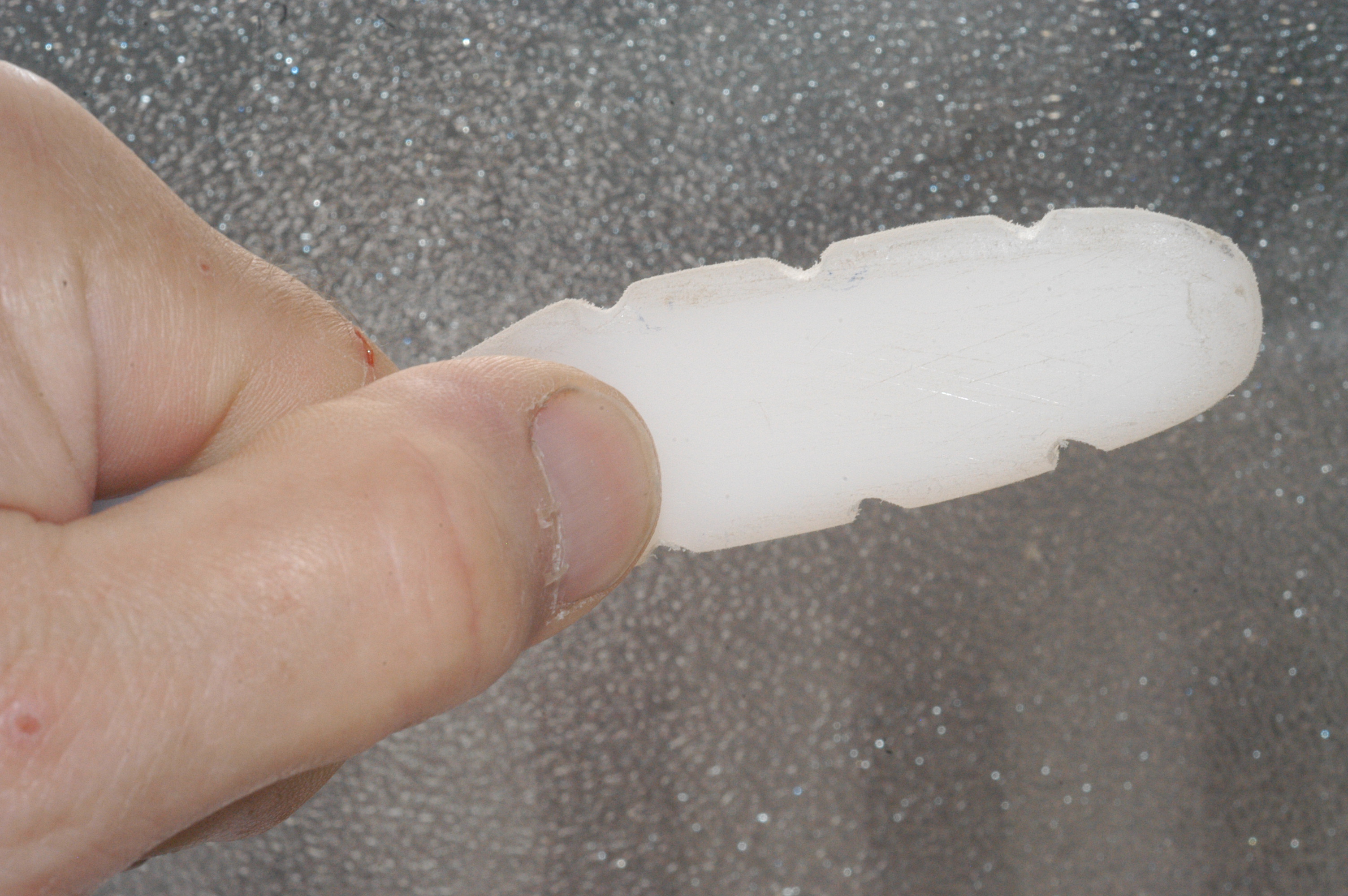


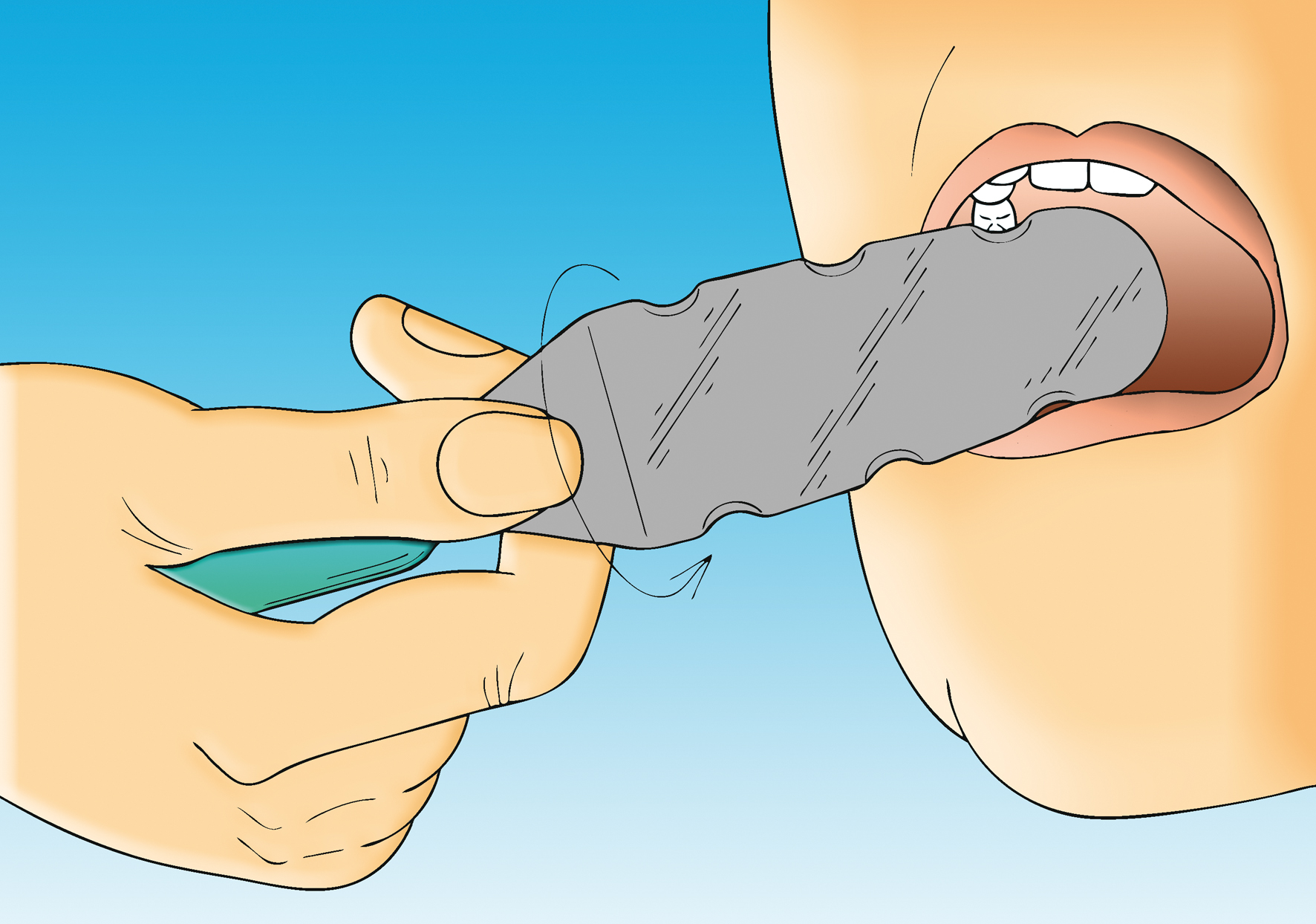


## Annexe 4 : notice d’utilisation de la SAB – version N°2 du 15/01/2016

Spatule SAB

Notice d’utilisation

1. **Description et caractéristiques techniques.**

La Spatule SAB est un dispositif médical d’aide à l’accessibilité buccale. Elle facilite l’accès à la cavité buccale chez des personnes n’ayant pas la capacité de le faire spontanément. Elle est particulièrement adaptée aux personnes en situation de déficience ou de dépendance pour qui l’accès en bouche est rendu difficile du fait d’un trouble du comportement lié à la déficience ou la dépendance.

La Spatule SAB est composée d’un manche de préhension et d’une lame introduite en bouche. Le manche et la lame sont reliés entre eux par une partie intermédiaire formant un angle de décrochement facilitant l’introduction en bouche de la lame.

- Le manche est recouvert de stries de rétention pour permettre une bonne préhension
- La partie intermédiaire présente une zone de dépression servant de zone de placement du pouce
- La lame est de forme mousse, arrondie à son extrémité et présente des encoches symétriques sur ses bords latéraux afin de permettre à la lame de se caler entre les arcades en évitant un glissement de la spatule vers l’intérieur de la cavité buccale.

La spatule SAB est fabriquée par une technique de plasturgie. Elle est composée de Polypropylène à usage médical ayant des propriétés mécaniques compatibles avec les forces masticatoires mises en jeu d’une part et avec la nature des éléments dentaires en contact avec la spatule SAB d’autre part (elle est suffisamment résistante pour ne pas se briser et suffisamment souple pour ne pas léser les tissus durs).

1. **Indications.**

La Spatule SAB est indiquée dans les cas de difficultés d’accessibilité buccale liées à des troubles comportements de la personne.

1. **Contre-indications.**

La Spatule SAB n’est pas indiquée dans les cas de limitation pathologique de l’ouverture buccale.

1. **Précautions d’emploi.**

Comme tout dispositif de contention, la Spatule SAB doit être utilisée dans le respect de la personne. Particulièrement, son utilisation doit se faire sans utilisation de la force physique qui pourrait la rendre traumatique à un niveau physique et traumatisante à un niveau émotionnel.

1. **Effets indésirables.**

Il existe des possibilités de lésions traumatiques liées à l’utilisation de la Spatule SAB.

Ce sont essentiellement des risques induits :

- blessures muqueuses (labiale, gingivale, jugale, …)
- blessure linguale
- fracture dentaire
- luxation dentaire
- expulsion dentaire
- reflexes nauséeux et vomissements
- douleurs
- complications articulaires
- inhalation ou ingestion de fragments dentaires

1. **Mode d’emploi.**

La Spatule SAB est prise en main par l’utilisateur selon un mode classique. Le manche est placé dans la paume de la main. Le pouce est placé sur la zone marquée sur la partie intermédiaire, ce qui défini la position exacte de la prise en main de la spatule.

De part sa faible épaisseur, la lame peut être placée entre les arcades maxillaires et mandibulaires au niveau des secteurs prémolo-molaires. Elle est introduite dans la cavité buccale jusqu’aux premières encoches.

Un mouvement de rotation est alors exercé doucement sur le manche afin de permettre l’ouverture buccale. Cette dernière peut être ajustée par l’utilisation des autres encoches de la lame.

L’ouverture buccale peut être alors maintenue par l’utilisateur, ce qui permet un contrôle constant de l’accès à la cavité buccale.

L’accessibilité de la zone controlatérale de la cavité buccale est alors possible.

La reproduction de cette procédure du coté opposé permettra de la même manière l’accès de la zone controlatérale de la cavité buccale.


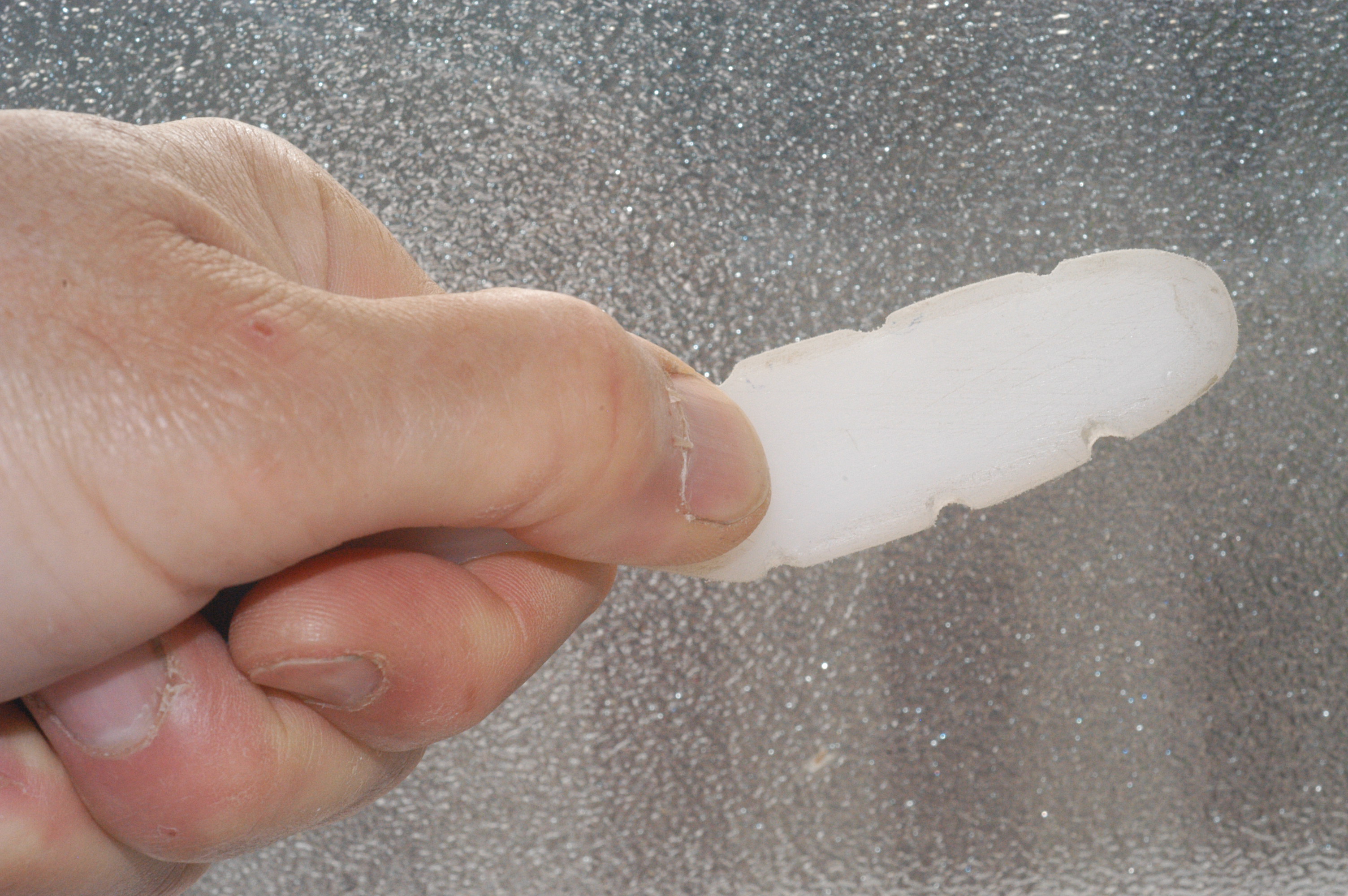


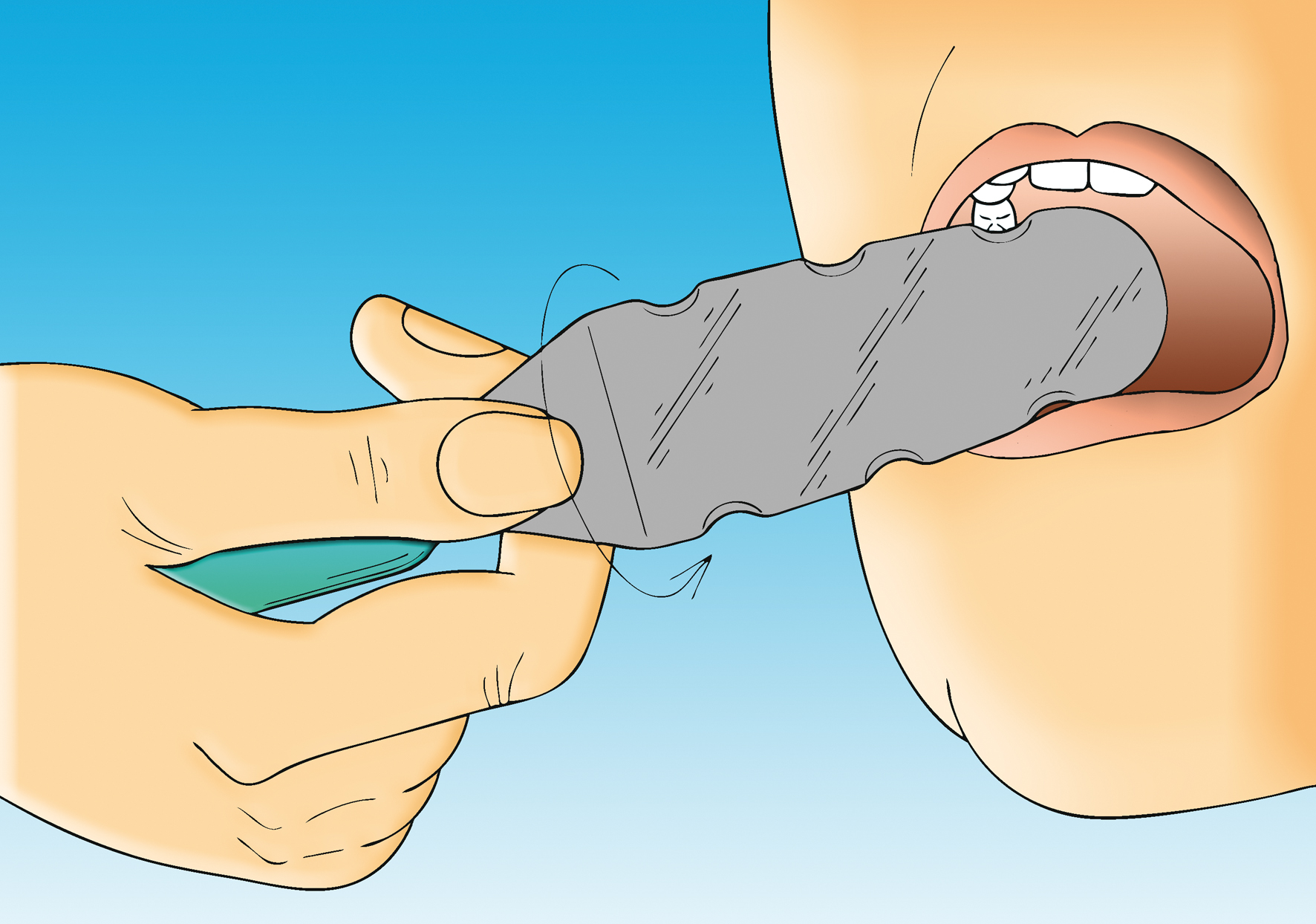


1. **Grossesse et allaitement.**

La Spatule SAB n’a pas été testée sur les femmes enceintes ou allaitantes.

1. **Conditionnement, stérilisation.**

La Spatule SAB est à usage unique.

Elle sera conditionnée en double emballage stérile selon un protocole de stérilisation ~~hospitalière classique (134°C à 5 bars de pression).~~ pour usage hospitalier par radiations ionisantes aux rayonnements gamma (25 kGy).

Elle peut être conservée à température ambiante.

## Annexe5 : fiche technique du polypropylène

##

##

## Annexe 6 : Echelle de Venham

0 *Détendu*, souriant, ouvert, capable de converser, meilleures conditions de travail possibles. Adopte le comportement voulu par le dentiste spontanément, ou dès qu’on le lui demande.

1 *Mal à l’aise*, préoccupé. Regard direct mais expression faciale tendue. Observe furtivement l’environnement. S’appuie spontanément sur le dossier du fauteuil. Les mains restent baissées ou sont partiellement levées pour signaler l’inconfort. Pendant une manœuvre stressante, peut protester brièvement et rapidement pour montrer son inconfort. Le patient est disposé à – et capable de – dire ce qu’il ressent quand on le lui demande. Respiration parfois retenue. Capable de bien coopérer avec le dentiste.

2 *Tendu*. Le ton de la voix, les questions et les réponses traduisent l’anxiété. Multiplie les demandes d’informations. Mains crispées aux accoudoirs, peuvent se tendre et se lever, mais sans gêner le dentiste, s’appuie au dossier spontanément, mais la tête et le cou restent tendus. Accepte la main –dans–la – main. Regard direct. Pendant une manœuvre stressante, protestations verbales, pleurs discrets possibles. Le patient interprète la situation avec une exactitude raisonnable et continue de maitriser son anxiété. Les protestations sont plus gênantes. Le patient obéit encore lorsqu’on lui demande de coopérer. La continuité thérapeutique est préservée.

3 *Réticent* à accepter la situation thérapeutique, à du mal à évaluer le danger. Soupire souvent. Protestations énergiques, pleurs possibles. S’appuie au dossier après plusieurs sollicitations, la tête et le cou restent tendus. Légers mouvements d’évitement. Mains crispées, regard parfois fuyant. Accepte la main –dans–la – main. Hésite à utiliser les mains pour essayer de bloquer les gestes du dentiste. Gigote un peu. Proteste verbalement, larmoyant. Protestations sans commune mesure avec le danger ou exprimées bien avant le danger. Parvient à faire face à la situation, avec beaucoup de réticence. La séance se déroule avec difficulté.

4 *Très perturbé* par l’anxiété et incapable d’évaluer la situation. Crispation importante. Sourcils froncés, regard fuyant, les yeux peuvent être volontairement fermés. Pleurs véhéments sans rapport avec le traitement. Mouvements d’évitement brusques. Pose ses mains sur sa bouche ou sur le bras du dentiste mais finit par se laisser faire. Serre les lèvres mais finit par garder la bouche ouverte. Soulève fréquemment sa tête du dossier. Rejette le contact corporel, mais peut encore accepter le main – dans – la – main. Importantes contorsions, nécessitant parfois une contention. Le patient peut être accessible à la communication verbale et finir, après beaucoup d’effort et non sans réticence, à essayer de se maitriser. La dissociation est partielle. La séance est régulièrement interrompue par les protestations.

5 *Totalement déconnecté* de la réalité du danger, inaccessible à la communication. Rejette le contact corporel. Serre les lèvres et les dents. Referme la bouche et serre les dents dès que possible. Agite violemment la tête. Pleure fort à grands cris, hurle, dit des injures, se débat, est agressif ; inaccessible à la communication verbale et visuelle. Quel que soit l’âge, présente des réactions primitives de fuite. Tente activement de s’échapper. Contention indispensable.

## Annexe 7: fiche de déclaration des EIG

|  | | **Réservé promoteur** | | | | | | | | | | | | |
| --- | --- | --- | --- | --- | --- | --- | --- | --- | --- | --- | --- | --- | --- | --- |
|  |  | **Fiche de NOTIFICATION**  **D’EVENEMENT INDESIRABLE GRAVE**  **à faxer dûment complétée dans les 24 heures ouvrées au 03 87 55 77 64** | | | | | | | | | | | | |
| **SAB : Evaluation de l’efficacité et de la sécurité d’emploi d’un dispositif innovant permettant d’améliorer l’accessibilité buccale des personnes en situation de handicap présentant des troubles du comportement : la Spatule d’Accessibilité Buccale.**  **N° ID RCB : 2012-a01535-38**  **Investigateur principal :** Dr Daniel ANASTASIO  Service d’Odontologie, CHR METZ-THIONVILLE - Hôpital Bel Air  1-3 rue du Friscaty – BP 60327 – 57126 THIONVILLE cedex  Tel : 0382558169– Mail : d.anastasio@chr-metz-thionville.fr | | | | | | | | | | | 1^ère^ notification  Date _ _ / _ _ / _ _ _ _    Rapport de suivi n° : |_|  Date _ _ / _ _ / _ _ _ _ | | | |
| **Patient N° Inclusion |__|__|__| Initiales : |__|-| __|**  Date de naissance : _ _ / _ _ / _ _ _ _ Age (ans) :  Poids (kg) : |__|__|__| Taille (cm) : |__|__|__|  Date inclusion dans l’étude : _ _ / _ _ / _ _ _ _ | | | | | | | | **Critère de gravité**  décès  mise en jeu du pronostic vital  nécessité d’hospitalisation ou de prolongation d’hospitalisation  invalidité/ incapacité temporaire ou permanente  anomalie, malformation congénitale  autres évènements médicalement significatifs | | | | | | |
| **Désignation de l’EIG :** | | | | | | | | | | | | | | |
| **Causalité établie par l’investigateur :**  Evènement lié au dispositif médical expérimental  Evènement non lié au dispositif médical expérimental  Ne peut se prononcer  **Médicaments concomitants** (à l’exclusion de ceux utilisés pour traiter l’événement)  si nécessaire compléter avec une autre feuille | | | | | | | | | | | | | | |
| **Nom** | **Forme** | | | **Voie** | | **Posologie** | | | **Date de début** | | | | **Date de fin** | |
|  |  | | |  | |  | | | _ _ /_ _ /_ _ _ _ | | | | _ _ /_ _ /_ _ _ _ | |
|  |  | | |  | |  | | | _ _ /_ _ /_ _ _ _ | | | | _ _ /_ _ /_ _ _ _ | |
|  |  | | |  | |  | | | _ _ /_ _ /_ _ _ _ | | | | _ _ /_ _ /_ _ _ _ | |
|  |  | | |  | |  | | | _ _ /_ _ /_ _ _ _ | | | | _ _ /_ _ /_ _ _ _ | |
|  |  | | |  | |  | | | _ _ /_ _ /_ _ _ _ | | | | _ _ /_ _ /_ _ _ _ | |
|  |  | | |  | |  | | | _ _ /_ _ /_ _ _ _ | | | | _ _ /_ _ /_ _ _ _ | |
|  |  | | |  | |  | | | _ _ /_ _ /_ _ _ _ | | | | _ _ /_ _ /_ _ _ _ | |
| Antécédents médicaux et chirurgicaux : | | | | | | | | | | | | | Dates | |
| **Etude SAB 2012-A01535-38** | | | | | **Réservé promoteur** | | | | | | | | |  |
| N° Inclusion |__|__|__| Initiales : |__|-| __| | | | | | | | | | | | | | |  |
| **Description de l’évènement indésirable grave**:  Date de début : _ _ / _ _ / _ _ _ _ heure : |__|__| H |__|__| min  Délai de survenue après la mise en œuvre du dispositif médical expérimental :   heures  jours  mois  Description : *Diagnostic ou symptômes prédominants. Décrire la chronologie de l’événement et les mesures thérapeutiques qui ont été prises*.  **Comptes-rendus anonymisés d'hospitalisation d'examens et/ou résultats de laboratoire joints :** **oui** **non** | | | | | | | | | | | | | |  |
| Action prise | | | | | | | | | | | | | |  |
| Aucune Recours à une action thérapeutique : | | | | | | | | | | | | | |  |
| **Évolution**  guérison sans séquelle Date de la guérison : _ _ / _ _ / _ _ _ _  guérison avec séquelles : _ _ _ _ _ _ _ _ _ _ _ _ _ _ _ _ _  sujet non encore rétabli   inconnue | | | | | | | **En cas de décès** : date du décès : _ _ / _ _ / _ _ _ _  décès sans rapport avec l’effet  décès auquel l’effet a pu contribuer  décès dû à l’effet | | | | | | |  |
| **Nom de l’investigateur** | | | | | **Date** | | | | | **Signature** | | | |  |
| **Cadre réservé au promoteur** Date de réception_ _ /_ _ /_ _ _ _ | | | | | | | | | | | | | |  |
|  | | | **Causalité** Evènement lié à l’étude  Evènement non lié à l’étude  Ne peut se prononcer | | | | | | | | | **L’effet indésirable est**  Attendu  Inattendu | |  |
| Commentaires : | | | | | | | | | | | | | |  |
| Demande information complémentaire oui non | | | | | | | | | | Signature | | | |  |

**ANNEXE 8: GRILLE OPTIMON**

**Définition du niveau de risque de la recherche pour la sécurité du patient**(à partir de la grille OPTIMON)

| **THEME D’ETUDE** | | | | | | | |
| --- | --- | --- | --- | --- | --- | --- | --- |
|  | | | | | | | |
| **ESSAI CLINIQUE DE MEDICAMENT RADIOTHERAPIE**  **THERAPIE GENIQUE**  **OU CELLULAIRE** | **CHIRURGIE** | **DISPOSITIF MEDICAL**  **dont IMAGERIE,**  **RADIOLOGIE, RADIO-ISTOPES** | **RISQUE DE L’ETUDE** | | | **PHYSIOPATHOLOGIE GENETIQUE AUTRES INTERVENTIONS** | **QUESTIONNAIRE QUALITE DE VIE PSYCHIATRIE** |
| --- | Technique peu invasive | Marqué CE, de classe I ou IIa, en routine  Marqué CE, de classe I, hors indication | 🡺 | A | 🡸 | Peu ou pas invasif  (dont prise de sang)  non contraignant | Questionnaire sans difficultés particulières |
| Etude confirmatoire sur produit avec autorisation ou nouvelle association et utilisation conforme à l’AMM | Technique ou biopsie sur organe interne | Marqué CE, de classe IIa, hors indication  Marqué CE, de classe IIb ou III, en routine  Non marqué CE, de classe I, non invasif et non  actif | 🡺 | B | 🡸 | Invasif ou contraignant | Questionnaire destabilisant dans une pathologie grave |
| Etude confirmatoire sur produit avec autorisation et utilisation différente de l’AMM  Etude exploratoire sur produit avec autorisation ou nouvelle association | Généralisation d’une nouvelle technique | Marqué CE, de classe IIb, hors indication  Marqué CE, de classe IIb ou III, avec peu de recul | 🡺 | C | **CONDITIONS D'AUGMENTATION DU RISQUE**  **a) INTERVENTION A RISQUE**, dont risque de mortalité ou de morbidité sévère liée à l'intervention, nouvelle indication, sevrage potentiellement dangereux, acte invasif avec pénétration par une autre voie qu'un orifice du corps (hors prise de sang)  **b) INVESTIGATION A RISQUE**, dont risque de mortalité ou de morbidité sévère liée à l'investigation, utilisation d’un produit radioactif, ou peu connu, ou sans autorisation, acte invasif avec pénétration par une autre voie qu'un orifice du corps (hors prise de sang)  **c) POPULATION A RISQUE**, dont risque de mortalité ou de morbidité sévère liée à une pathologie grave ou à l'âge, défaillance ou insuffisance d’un système ou organe, âge ≤ 2 ans, âge ≥ 80 ans, femme enceinte, parturiente ou allaitante |  |  |
| Etude exploratoire sur produit sans autorisation  1ères études sur l’homme (pharmacologie, bioéquivalence) | Mise au point d’une nouvelle technique | Marqué CE, de classe III, hors indication  Non marqué CE, de classe I, invasif ou actif  Non marqué CE, de classe IIa ou IIb ou III | 🡺 | D |  |  |  |


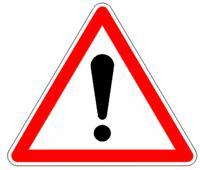
 Si la grille OPTIMON est utilisée, l’augmentation du risque liée aux populations particulières

(loi 2004-806, cf page 4) n’est pas comptée pour le score LI.

**ANNEXE 9 : Calcul du score « Logistique, Impact, Ressources»  pour les recherches (A, B, C)**

Après l’évaluation du risque patient lié à la participation à la recherche celui-ci est pondéré par les considérations d’impact et de stratégie de la recherche.

|  | | | | | | ***Reporter le score correspondant*** |
| --- | --- | --- | --- | --- | --- | --- |
| **SCORE LOGISTIQUE** | **Caractère mono/ multicentrique** | **Monocentrique** | | | 0 |  |
|  |  | **Multicentrique** | | - Interrégional - National - International | 1  2  3 | 1 |
|  |  |  |  | - 1 à 5 centres extérieurs - 6 à 10 centres extérieurs - 11 à 20 centres extérieurs - > 20 centres | 1  2  3  4 | 1 |
|  | **Complexité Logistique** | **Circuit Biologie** | | - Non - Oui | 0  1 | 0 |
|  |  | **Circuit Imagerie** | | - Non - Oui | 0  1 | 0 |
|  |  | **Circuit Produits** | | - Non - Oui | 0  1 | 1 |
|  |  | **Circuit Patient** | | - Non - Oui | 0  1 | 0 |
|  |  | **Circuit Données** | | - Non - Oui | 0  1 | 0 |
|  | **Nombre de patients attendus** | | | - < 50 - 50 – 200 - > 200 | 0  1  2 | 1 |
|  | **Durée de participation par patient** | | | - < 7 jours - 7 jours – 6 mois - 6 mois – 2 ans - > 2 ans | 0  1  2  3 | 0 |
|  | **CRF** | | **Type CRF** | - e-CRF - Papier | 0  1 | 1 |
|  |  |  | **Nombre de pages ou variables** | - < 20 pages ou < 200 variables - de 20 à 50 pages ou 200 à 500 - > 50 pages ou > 500 | 0  1  2 | 0 |
|  | **SCORE LOGISTIQUE** | | | | | **5 / 20** |
| **SCORE IMPACT** | **Schéma de l’Etude** | **Randomisée** | | - Non - Oui | 0  1 | 1 |
|  |  | **Recherche en insu** | | - Non - Oui | 0  1 | 0 |
|  | **Fragilité des données** | **Pathologie rare** | | - Non - Oui | 0  1 | 0 |
|  |  | **Pédiatrie, population particulière*** | | - Non - Oui | 0  1 | 1 |
|  |  | **Maladie saisonnière** | | - Non - Oui | 0  1 | 0 |
|  |  | **Situation d’urgence / Réanimation** | | - Non - Oui | 0  1 | 0 |
|  | **Recueil de données sensibles** | | | - Non - Oui | 0  1 | 0 |
|  | **Impact des résultats** | | | - Autre / Publication - Etude médico-éco / STIC - Faite à la demande des autorités | 0  1  2 | 0 |
|  | **Impact / risque médiatique**  **et/ou politique** | | | - Non - Oui | 0  1 | 0 |
|  | **Brevet potentiel** | | | - Non - Oui | 0  1 | 1 |
|  | **Etude pouvant être intégrée dans un dossier d’AMM (ou extension)** | | | - Non - Oui | 0  1 | 0 |
|  | **Financement DGOS, ANR, Europe, Inca** | | | - Non - Oui | 0  1 | 0 |
|  | **Contrat de partenariat industriel ou autre** | | | - Non - Oui | 0  1 | 0 |
|  | **SCORE IMPACT** | | | | | **3 / 14** |
| **SCORE RESSOURCES** | **Expérience investigateur coordonnateur** | | | - Expérience coordonnateur - Expérience d’investigateur principal (pas coordonnateur) - Ni comme coordonnateur ni investigateur principal | 0  1  2 | **2** |
|  | **Expérience investigateur centres associés** | | | - > 50% des centres - < 50% des centres | 0  1 | **1** |
|  | **Présence de personnel de recherche dans le centre coordonnateur** | | | - Expérience coordination d’étude - Expérience d’investigation - Non ou sans expérience | 0  1  2 | **0** |
|  | **Présence de personnel de recherche dédié dans les centres associés** | | | - > 50% des centres - < 50% des centres | 0  1 | **1** |
|  | **SCORE RESSOURCES** | | | | | **4 / 6** |
| **SCORE TOTAL LOGISTIQUE + IMPACT + RESSOURCES** | | | | | | **12/ 40** |

**Définition du niveau de monitoring en fonction du risque patient et du score « LIR »**

| **Risque patient** | **Score total « Logistique Impact Ressources »** | |
| --- | --- | --- |
|  | **De 1 à 19** | **>20** |
| **A** | Niveau minimal | Niveau intermédiaire |
| **B** | Niveau minimal | Niveau intermédiaire |
| **C** | Niveau intermédiaire | Niveau élevé |
| **D** | Niveau élevé | |
