# Supplementary material for: Efficacy, tolerability, and safety of an innovative medical device for improving oral accessibility during oral examination in special-needs patients: A multicentric clinical trial
Source: PLoS One. 2020 Sep 28;15(9):e0239898. doi: 10.1371/journal.pone.0239898 (PMC7521731; doi:10.1371/journal.pone.0239898)
Supplement: Attachment — Submitted filename: renamed_fd776.docx [file pone.0239898.s007.docx]

**COMMENTS – Manuscript PONE-D-20-09935**

Title: “Efficacy, tolerability, and safety of an innovative medical device for improving oral accessibility during oral examination in patients with special needs: A multicentric clinical trial”

**Important note: This review pertains only to ‘statistical aspects’ of the study and so** **‘****clinical aspects’ [like medical importance, relevance of the study,** **‘clinical significance and implication(s)’ of the whole study, etc.] are to be evaluated [should be assessed] separately/independently. Further please note that any ‘statistical review’ is generally done under the assumption that (such) study specific methodological [as well as execution] issues are perfectly taken care of by the investigator(s). This review is not an exception to that and so does not cover clinical aspects {however, seldom comments are made only if those issues are intimately / scientifically related & intermingle with ‘statistical aspects’ of the study}.**

**COMMENTS:**

You should have given few details [very brief] of ‘Venham test’ which, I guess, is based on eight ‘pictures’ & measures subject’s dental anxiety {maximum score 8}. As even other tools used are expected to yield data in ‘ordinal’ level of measurement, application of non-parametric test(s) [ex. Wilcoxon’s signed ranks test] was/were desirable anyway and followed here is good. However, please note that ‘Multiple linear regression’ used to identify the factors that are associated with spatula-induced variation in the ‘Total Oral Accessibility Score’ is parametric [whereas ‘logistic regression’ is semi-parametric, completely non-parametric regression is also available but not popular (& so no software available, computations are very cumbersome) and moreover, is not ‘powerful’, however, often the logistic regression is considered by many as non-parametric].

I am sure that the authors are aware of the well-known drawbacks of before-after study which is a single-arm design [a type of Quasi-experimental research, it is alright to conduct ‘single-arm’ (before-after) study when that is the only possibility]. It is very essential to keep those limitations in mind while interpreting results. Also note that a classical/ideal clinical trial/study needs/requires a concurrently handled/treated appropriated selected/chosen control/comparison parallel group/arm [pointed-out in limitations later].

I am not sure if present study be called as ‘crossover’ [line 165 in Methods - Study design section: This prospective multicenter interventional open-label one-way crossover clinical trial] when according to lines 217-220 “The study intervention consisted of two immediately successive oral examinations, first without the spatula and then with the spatula. The temporal order of the two oral examinations was fixed rather than being randomized because the spatula should only be used if there is difficulty obtaining access to the oral cavity”. If the word/term ‘one-way crossover’ clinical trial’ is planted/used here to indicate this phenomenon then it is expected to be clarified that way because the phrase ‘one-way crossover clinical trial’ is new (to most including me). When you use a word ‘crossover’ to describe a clinical trial design, a particular/typical trial design comes to mind which is entirely different than the present one.

As per account in lines 219-20 i.e. spatula should only be used if there is difficulty obtaining access to the oral cavity, shows that action later depends on outcome of first. And yet the design is called crossover. Only one variation is known [to/in the literature], called as ‘case-crossover’ design [a 'case window', the period of time during which the person was assumed to be a case, and a 'control window’ otherwise] and if that is what it meant {some similarity}, it should be clarified. If this one is proposed as new variation, please explain it in more details. {There was one paper describing/using ‘wait-listed’ control as cross-over but had given details like consequences on analyses}

It is highly appreciable that a measure called ‘Oral Accessibility Score’, which is also the primary endpoint of the study, is/ was newly devised for this study. But note that it is mandatory to study/establish its ‘psychometric properties’ [like validity, reliability] after new development / before use. Same is with the ‘Examiner Satisfaction Scale’ Likert scale which was newly devised for this study. {Note that important properties like validity, reliability incidentally is/are been called ‘psychometric properties’ but have nothing to do with ‘psychology’}.

Estimation of sample size required/needed to detect the ability of the spatula to increase 20% from baseline frequency of 20%, is correct [please make the sentence little simple to understand more clearly]. All the tools used also seems appropriately used / alright [except for properties of few].

What is the interpretation of values [parameter estimate, Standard deviation & ‘P’ for each “Factor”] displayed in table-4? Is not this a natural question in anybody’s mind? Will you please explain them little bit? Why divided (what is the purpose of dividing) ‘factor’ into categories [like Age as 0-12, 13-17, 18-64, ≥65]? According to my knowledge, nature [type & level of measurement] even of independent variables (in addition to dependent variable) matters in ‘Multiple linear regression’ [all variables are treated as numerical/continuous so that Sex (female=1, male=2) is different than (male=1, female=2)] . Why ‘Center no.1’ is taken as ‘reference category’? {this info may add to understanding, hopefully}. I guess only in logistic regression (and not in ‘Multiple linear regression’) it is necessary to identify ‘reference category’ [if there are more categories than two for a variable] because ‘odds ratio’ is estimated with reference to that category. If those reported ‘parameter estimate’s [column 2 of table 4] are ‘odds ratios’ {i.e. if binary logistic regression is used} then why not report two categories made of dependent variable (Total Oral Accessibility Score)?

Prerogative/claim mentioned in lines 400-402 [that: Consequently, it may be useful in not only dental practice but also for caregivers during toothbrushing, thereby helping to improve the parlous oral health of this population] is not clear. Will you please help me understand how it may be helping to improve the parlous oral health of this population?

Pointing-out all possible ‘limitations’ [lines 490-543; particularly highlighting that ‘potential bias could have been avoided by conducting a parallel-arm trial’ and ‘further studies validating this scale are needed’ is highly appreciated. In fact, most of the above review cover these issues only. Except these points, the article is very well drafted and study is excellently executed. Though I do not fully agree with positive features listed [lines 544-550], I can definitely say that this article should be accepted with above minor revisions suggested.
